# Transcriptomic and metabolomic profiling reveals the effect of LED light quality on morphological traits, and phenylpropanoid-derived compounds accumulation in *Sarcandra glabra* seedlings

**DOI:** 10.1186/s12870-020-02685-w

**Published:** 2020-10-15

**Authors:** Dejin Xie, Lingyan Chen, Chengcheng Zhou, Muhammad Waqqas Khan Tarin, Deming Yang, Ke Ren, Tianyou He, Jundong Rong, Yushan Zheng

**Affiliations:** 1 College of Forestry, Fujian Agriculture and Forestry University, Fuzhou, 350002 China; 2 College of Arts & College of Landscape Architecture, Fujian Agriculture and Forestry University, Fuzhou, 350002 China

**Keywords:** *Sarcandra glabra*, LED lights, Transcriptomic profiling, Metabolomic profiling, Phenylpropanoid biosynthesis

## Abstract

**Background:**

*Sarcandra glabra* is an evergreen and traditional Chinese herb with anti-oxidant, anti-bacterial, anti-inflammatory, and anti-tumor effects. Light is one of the most influential factor affecting the growth and quality of herbs. In recent times, the introduction of Light Emission Diode (LED) technology has been widely used for plants in greenhouse. However, the impact of such lights on plant growth and the regulatory mechanism of phenylpropanoid-derived compounds in *S. glabra* remain unclear.

**Results:**

The red LED light (RL) substantially increased the plant height and decreased the stem diameter and leaf area relative to the white LED light (WL), while the blue LED light (BL) significantly reduced the height and leaf area of *S. glabra*. According to transcriptomic profiling, 861, 378, 47, 10,033, 7917, and 6379 differentially expressed genes (DEGs) were identified among the groups of leaf tissue under BL (BY) vs. leaf tissue under RL (RY), BY vs. leaf tissue under WL (WY), RY vs. WY, root tissue under WL (WG) vs. WY, stem tissue under WL (WJ) vs. WG, and WJ vs. WY, respectively. We identified 46 genes encoding for almost all known enzymes involved in phenylpropanoid biosynthesis, e.g., phenylalanine ammonia lyase (PAL), chalcone synthase (CHS), and flavonol synthase (FLS). We found 53 genes encoding R2R3-MYB proteins and bHLH proteins, respectively, where several were related to flavonoids biosynthesis. A total of 454 metabolites were identified based on metabolomic profiling, of which 44, 87, and 296 compounds were differentially produced in WY vs. RY, WY vs. BY, and WY vs. WG. In BY there was a substantial reduction in the production of esculetin, caffeic acid, isofraxidin, and fraxidin, while the yields of quercitrin and kaempferol were significantly up-regulated. In RY, the contents of cryptochlorogenic acid, cinnamic acid, and kaempferol decreased significantly. Besides, in WG, the production of metabolites (e.g. chlorogenic acid, cryptochlorogenic acid, and scopolin) declined, while their yields increased significantly (e.g. esculetin, fraxetin, isofraxidin, and fraxidin).

**Conclusion:**

These results provide further insight into the regulatory mechanism of accumulation patterns of phenylpropanoid-derived compounds in *S. glabra* under various light conditions, allowing optimum breeding conditions to be developed for this plant.

## Background

*Sarcandra glabra* (Thunb.) Nakai (Caoshanhu or Zhongjiefeng in Chinese medicine), belonging to the Chloranthaceae family, is an evergreen and traditional Chinese herb. *S. glabra* is widely distributed in Southern China and throughout Southeast Asia, mainly propagated by seeds or asexual methods (vegetative propagation) [1, 2]. The Pharmacopoeia of the People’s Republic of China has reported that the whole dry plant can be used as medicine [3], which contains abundant bioactive phytochemicals that not only contribute to protecting the plant against abiotic or biotic stress [4], but also confer anti-oxidant [5], anti-bacterial [6], anti-inflammatory [7], and anti-tumor [8] properties. Previous studies of this species mainly focused on the isolation and extraction of its phytochemical components, which can be divided into six groups; organic acids [9, 10], flavonoids [9], coumarins [2], terpenoids [11], phenolic acids [2, 5], and polysaccharides [1]. Among these components, flavonoids (e.g., quercitrin and kaempferol), coumarins (e.g., isofraxidin and scopoletin), and phenolic acids (e.g., rosmarinic acid) are closely associated with the phenylpropanoid biosynthesis.

To address the limited natural resources and meet consumer demand for safe and high-quality herbal products, China has initiated the project; “Good Agricultural Practice (GAP)” based on Chinese materia medica to enhance herb’s resources and quality of cultivation. Currently, various studies are underway to overcome the issue and challenges faced by the herbal industry in China, to improve the quality and growth rate of the medicinal plants, such as stereoscopic cultivation [12] or greenhouse cultivation [13]. Consequently, the production of *S. glabra* is gradually shifting from conventional models to more advanced and sustainable farming practices, such as greenhouse cultivation*.* Greenhouse cultivation is considered as an effective agricultural method for growing large quantities of herbs that help to ensure the product quality [14].

Environmental factors like water, light, temperature, and soil nutrients are key elements for the growth, development, and high quality of plants. Among these factors, it is necessary to emphasize the light factor because it is responsible for photosynthesis. Recently, the new-generation of Light Emission Diode (LED) technology has been applied to the greenhouse cultivation, providing favorable conditions for better plant growth and development [15, 16].

Recently, a large number of studies have shown that specific LED lights have a significant effect on plant morphogenesis [17–19]. For instance, both the stem length and the leaf area of the tomato seedlings were significantly restricted at the higher BL/RL ratio [19, 20]. Similarly, the hypocotyl length and leaf area were suppressed when cucumber seedlings were exposed to a high percentage of BL [21]. In addition, the different LED lights have shown a significant impact on the yields of the primary and species-specific secondary metabolites in plants. For instance, the BL significantly increased the yields of phenolic compounds in pea sprouts and basil [22, 23], flavonoids production in *Cyclocarya paliurus* [24], and the amount of salidroside in callus culture of *Rhodiola imbricata* [25]. On the contrary, the red and far-red parts of the light spectrum induce the production of salicylic acid, which in turn increased the flavonoids accumulation in *Ginkgo biloba* [26]. Furthermore, it was found that yellow LED light had a beneficial effect on the concentrations of flavonoids in *Epimedium pseudowushanense* [27]. Collectively, these studies have shown that flavonoids and other phenolic compounds originating from the phenylpropanoid pathway are accumulated in response to monochromatic LED light irradiation. The phenylpropanoid pathway is also responsible for the production of coumarins and lignins [28].

So far, many efforts have been made in elucidating the phenylpropanoid biosynthesis and the finding have shown that this complex regulatory network is highly dependent on several factors, including enzymatic interactions, transcription factors (TFs), and environmental factors [28, 29]. For TFs, MYB, bHLH, WDR, and the MBW complex (comprising of MYB, bHLH, and WD) are well conserved in plants and play a critical role in the regulation of the phenylpropanoid pathway [30–34].

The purpose of this study was to experimentally compare the effects of WL, RL, and BL on *S. glabra* seedlings, and analyze the transcriptomic and metabolomic profiling within these treatments. Besides, we also identified the functional genes and TFs (R2R3-MYB and bHLH) involved in the biosynthesis of phenylpropanoid-derived compounds, as well as their expression patterns. The findings of this study will provide a better understanding of the effects of the different monochromatic LED lights on plant growth and secondary metabolites accumulation*,* and promote the quantity and quality of *S. glabra* cultivation by fine-tuning the optimum light condition.

## Results

### Effects of different light qualities on plant growth

**Fig. 1:**
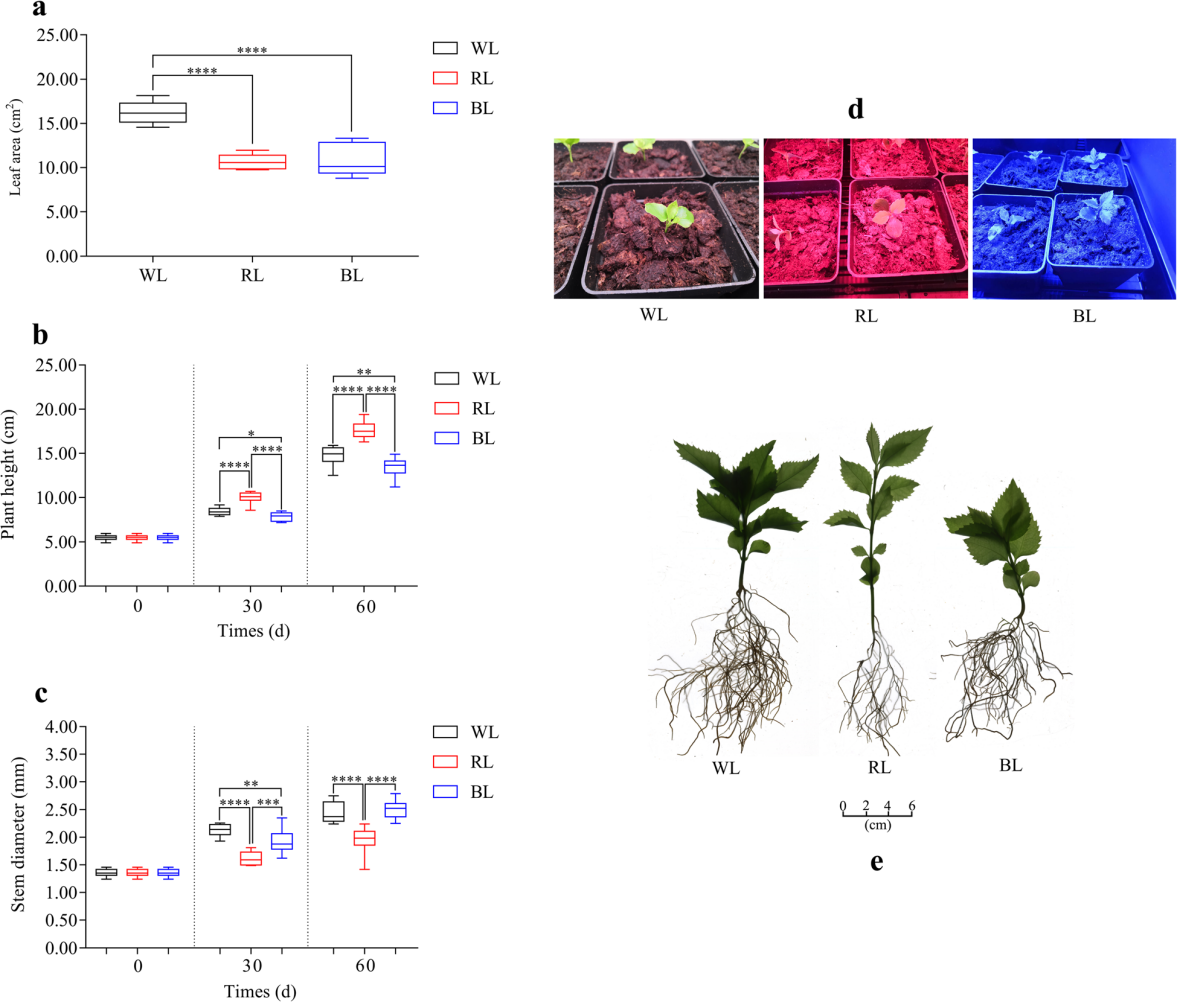
Images of the effect of LED lights on leaf area, plant height, and stem diameter. **a** the leaf area of the seedlings on the 60th day; **b** the plant height; **c** the stem diameter; **d** the *S. glabra* seedlings grown under different LED lights; **e** the whole plant morphology under WL, RL, BL; Maximum and minimum values are represented at the upper and lower ends of the whisker, respectively. The 75th and 25th percentiles are represented at the upper and lower ends of the box, respectively. * Represent *P* < 0.05, ** Represent *P* < 0.01, *** Represent *P* < 0.001, **** Represent *P* < 0.0001. WL: White LED light; RL: Red LED light; BL: Blue LED light

At the final harvest (day 60), the leaf area was significantly lower in the RL and BL treatments relative to the WL treatment (Fig. 1a). Compared to the WL, the plant height was higher in the RL treatment, whereas lower in BL treatments (Fig. 1b). On the 60th day, the stem diameters of plants under the WL and BL treatments were significantly greater than those in RL treatment (Fig. 1c). Details of statistical data have been presented in Additional file 1: Table S1.

### Illumina sequencing, de novo assembly, and annotation of the reference transcriptome

In total, 1025 million raw reads were generated from the 15 cDNA libraries with a total of 1009 million high-quality clean reads. Approximately 55.9–92.7 million raw reads were produced in each library (NCBI BioProject Accession: PRJNA664220, https://www.ncbi.nlm.nih.gov/bioproject/PRJNA664220). The relevant parameters of de novo assembly have been depicted in Additional file 2: Table S2. The results of the functional annotation from the seven public databases showed that 46.9% of unigenes were annotated in at least one database, while only 3.0% of unigenes were annotated in all the databases (Additional file 3: Table S3). A total of 33,626 unigenes were categorized into 56 functional groups among three categories: biological process, cellular component, and molecular function (Additional file 4: Fig. S1).

### DEGs analysis

Differential expression analysis of two groups was performed. The results showed that 861 (BY vs. RY), 378 (BY vs. WY), 47 (RY vs. WY), 10,033 (WG vs. WY), 7917 (WJ vs. WG), and 6379 (WJ vs. WY) genes were expressed significantly.

The enrichment analysis was performed using the GO ontology analysis (Additional file 5: Fig. S2). A large number of DEGs were predominant in five terms, namely metabolic process, single-organism metabolic process, catalytic activity, ion binding, and transferase activity. Meanwhile, all DEGs were mapped in the KEGG database (Additional file 6: Fig. S3). Except for RY vs. WY, in each group, the DEGs were enriched in the biosynthesis of phenylpropanoids (including flavonoids). The phenylpropanoid-derived compounds were just originated from this biosynthetic pathway. Therefore, the follow-up experiment and analysis were performed to describe this regulatory mechanism based on transcriptomic and metabolomic profiling.

### Analysis of metabolites

Based on the results obtained from the UPLC-MS/MS platform and self-compiled database, a total of 454 metabolites were detected, including various classes of lignans and coumarins, flavonoids, phenolic acids, and other. Moreover, the majority of them are phenylpropanoid-derived compounds, including flavonoids (90), phenolic acids (66), lignans and coumarins (11), and tannins (13) (Table 1 and Additional file 7: Table S4). Among them, the major ingredients of fraxetin, scopolin, isofraxidin, fraxidin, and esculin from the lignans and coumarins class and rosmarinic acid, rosmarinyl glucoside, chlorogenic acid, and esculetin from the phenolic acids class were isolated from *S. glabra* samples.

**Table 1:** Classes of the detected metabolites in *S. glabra*

Metabolite class	Number of metabolites	Metabolic class	Number of metabolites
Flavonoids	90	Organic acids	32
Alkaloids	39	Lipids	60
Phenolic acids	66	Tannins	13
Lignans and Coumarins	11	Terpenoids	10
Amino acids and derivatives	52	Others	53
Nucleotides and derivatives	28		

**Fig. 2:**
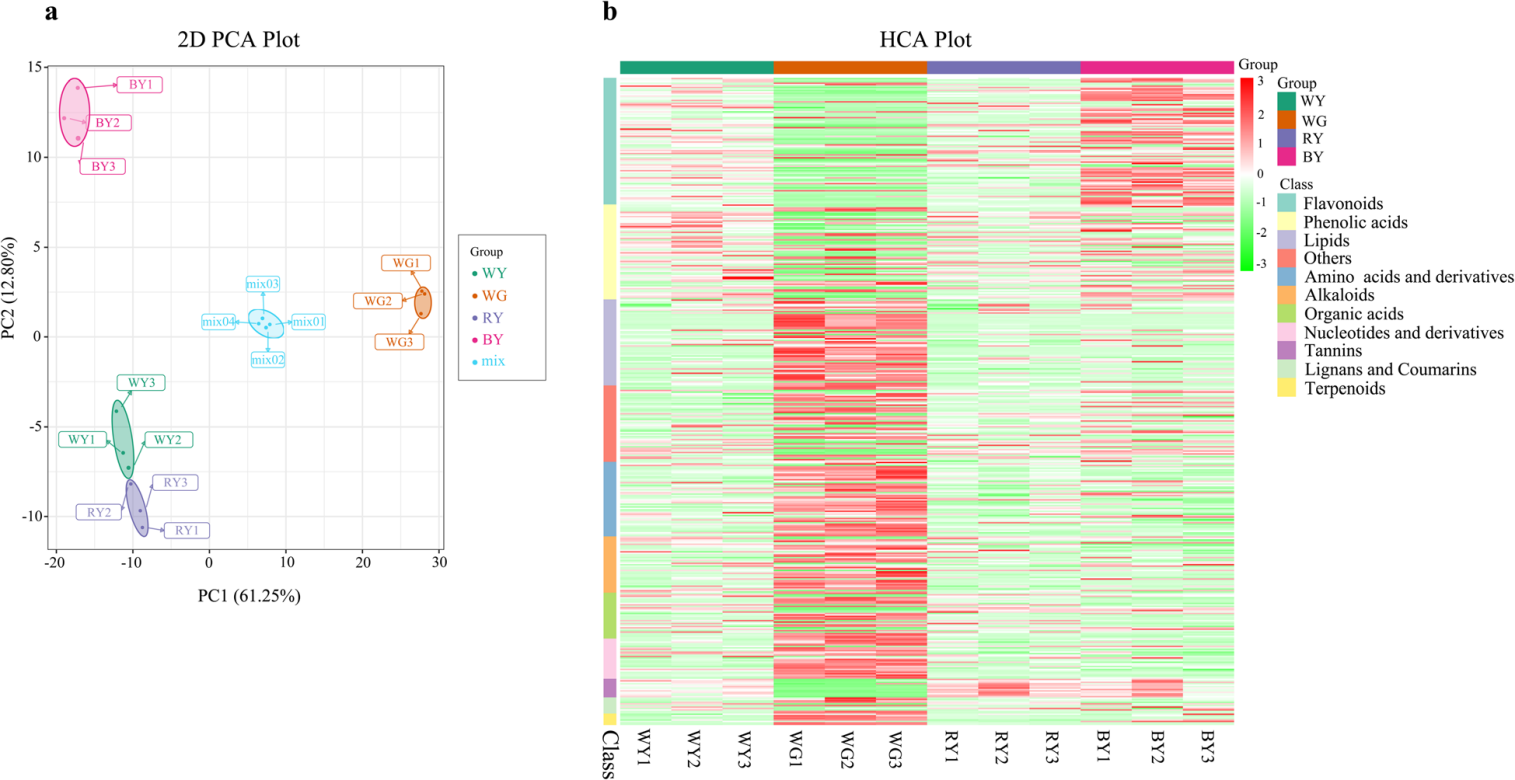
Results of PCA and HCA analyses of 12 samples from *S. glabra.* **a** In PCA plot, PC1 (61.25% variance) and PC2 (12.8% variance) were used to generate the scores plot. Samples of WY, RY, BY, WG, and mix are marked in green, purple, pink, brown, and blue, respectively. The mix sample is a quality-control sample that was made of an equal mixture of all samples. **b** In HCA plot, all the identified metabolites were divided into 11 classes, and each coloured rectangle represented the specific compound content. WY: Leaf tissue under white LED light; WG: Root tissue under white LED light; BY: Leaf tissue under blue LED light; RY: Leaf tissue under red LED light

The PCA (Fig. 2a) results showed that the tested samples were significantly different and their replication was generally consistent with that of others in the same group. The HCA (Fig. 2b) presented metabolic profiling and distributed them into 10 classes in all of the samples. Compared to WY and RY, most flavonoids contents (e.g. quercitrin and kaempferol) were higher in BY. Moreover, the distribution of chemicals was significantly different between the root (WG) and leaves (WY, RY, and BY) as a whole. Leaf tissues contained a higher abundance of compounds such as the classes of flavonoids, phenolic acids, and tannins, while the classes of lipids, amino acids and derivative alkaloids, organic acids, nucleotides and derivatives, lignans and coumarins, and terpenoids were much more abundant in the root tissue.

**Fig. 3:**
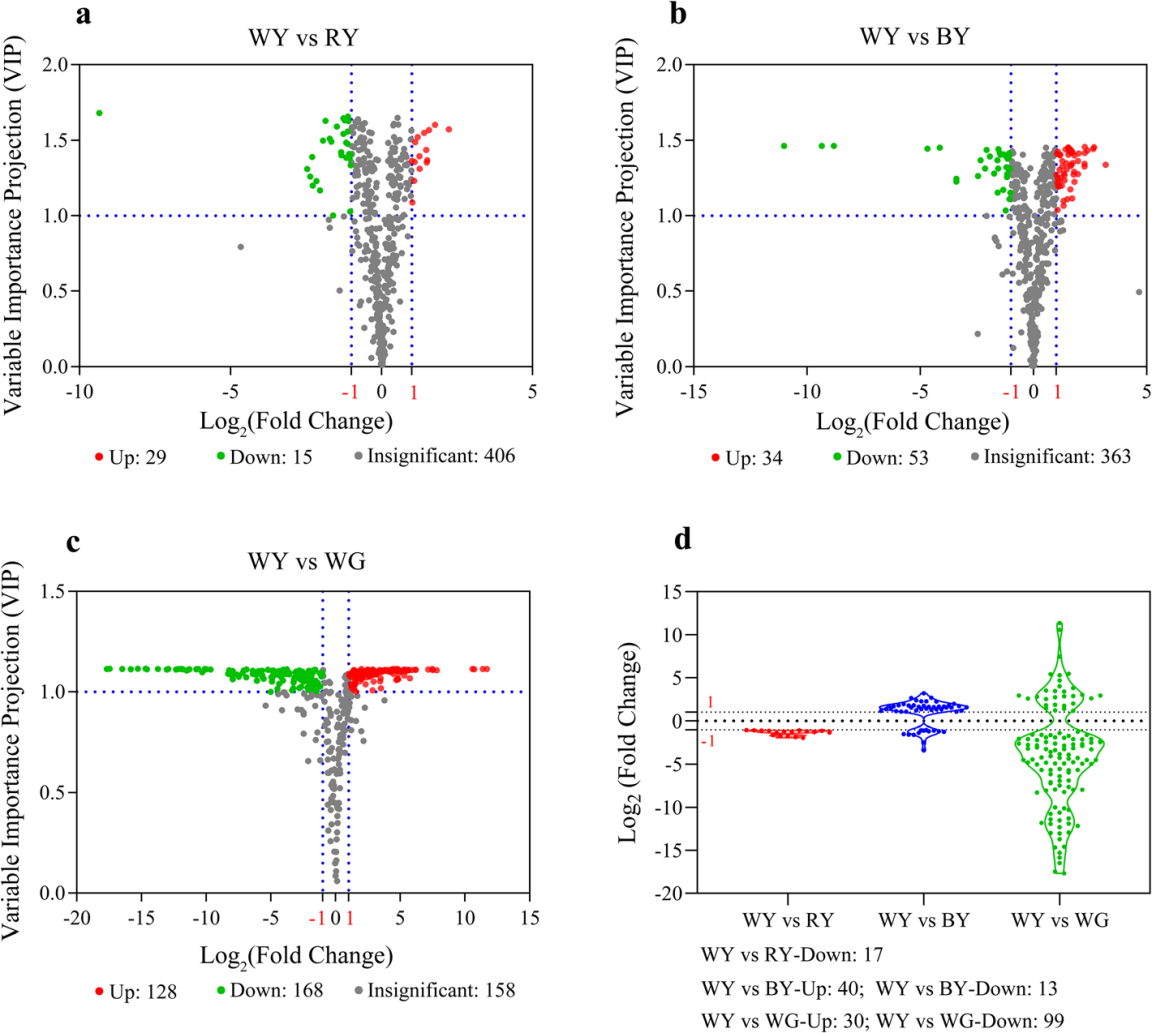
Metabolomic profiling of identified compounds in different groups. **a**, **b**, and **c** Volcano plots showing all the differential compounds in groups of WY vs. RY, WY vs. BY, and WY vs. WG. **d** Violin plot showing significantly differential phenylpropanoid-derived compounds in groups of WY vs. RY, WY vs. BY, and WY vs. WG. If VIP ≥ 1 and Log_2_(Fold change) ≥ 1 or ≤ − 1, they were regarded as the differential metabolites

The results of the comparative analysis are shown in Table 2 and Fig. 3. There were 44, 87, and 296 compounds that were differentially produced in WY vs. RY, WY vs. BY, and WY vs. WG, respectively (Fig. 3a, b, and c). Besides, compared to WY, 11 flavonoids (e.g., kaempferol) and 6 phenolic acids (e.g., cryptochlorogenic acid) were significantly down-regulated in RY (Table 2 and Fig. 3d). Among them, only 3 flavonols compounds (kaempferol, kaempferin, and cynaroside) were mapped in KEGG pathway related to flavonoids biosynthesis. In WY vs. BY group, 40 flavonoids, 11 phenolic acids, and 2 lignans and coumarins had significant differences. Among them, 40 of these compounds were up-regulated and 13 compounds were down-regulated (Table 2 and Fig. 3d). Moreover, in the flavonoid biosynthesis, only 8 out of 40 flavonoids were mapped, and 6 of these increased in BY by 3.2-to 6.2-fold, such as quercitrin and kaempferol. However, the contents of cynaroside and protocatechuic aldehyde were approximately reduced by 4.5-fold in BY. For phenolic acids, seven metabolites contents were down-regulated, including caffeic acid, esculetin, and sinapinaldehyde, whereas others increased by 2.5- to 3.3-fold. For lignans and coumarins compounds, in BY, daphnetin production decreased by 9.5-fold and oxypeucedanin increased by 2.1-fold. In terms of WY vs. WG group, 129 phenylpropanoid-derived metabolites were significantly up- or down-regulated (Table 2 and Fig. 3d). Within the group of WY vs. WG, compounds derived from the biosynthesis of flavonoids mainly accumulated in WY instead of WG (Fig. 2b). In contrast, some coumarins (esculin, isofraxidin, and fraxidin) were more abundant in WG, but the production of other coumarins (scopolin and oxypeucedanin) and most phenolic acids compounds were much higher in WY.

**Table 2:** The quantitative statistics of identified Phenylpropanoid-derived metabolites in different groups

Group name	Identified Metabolites	Phenylpropanoid-derived Metabolites	Phenylpropanoid-derived Metabolites (significant)	Up-regulated	Down-regulated
WY vs. RY	450	180	17	0	17
WY vs. BY	450	180	53	40	13
WY vs. WG	454	180	129	30	99

**Fig. 4:**
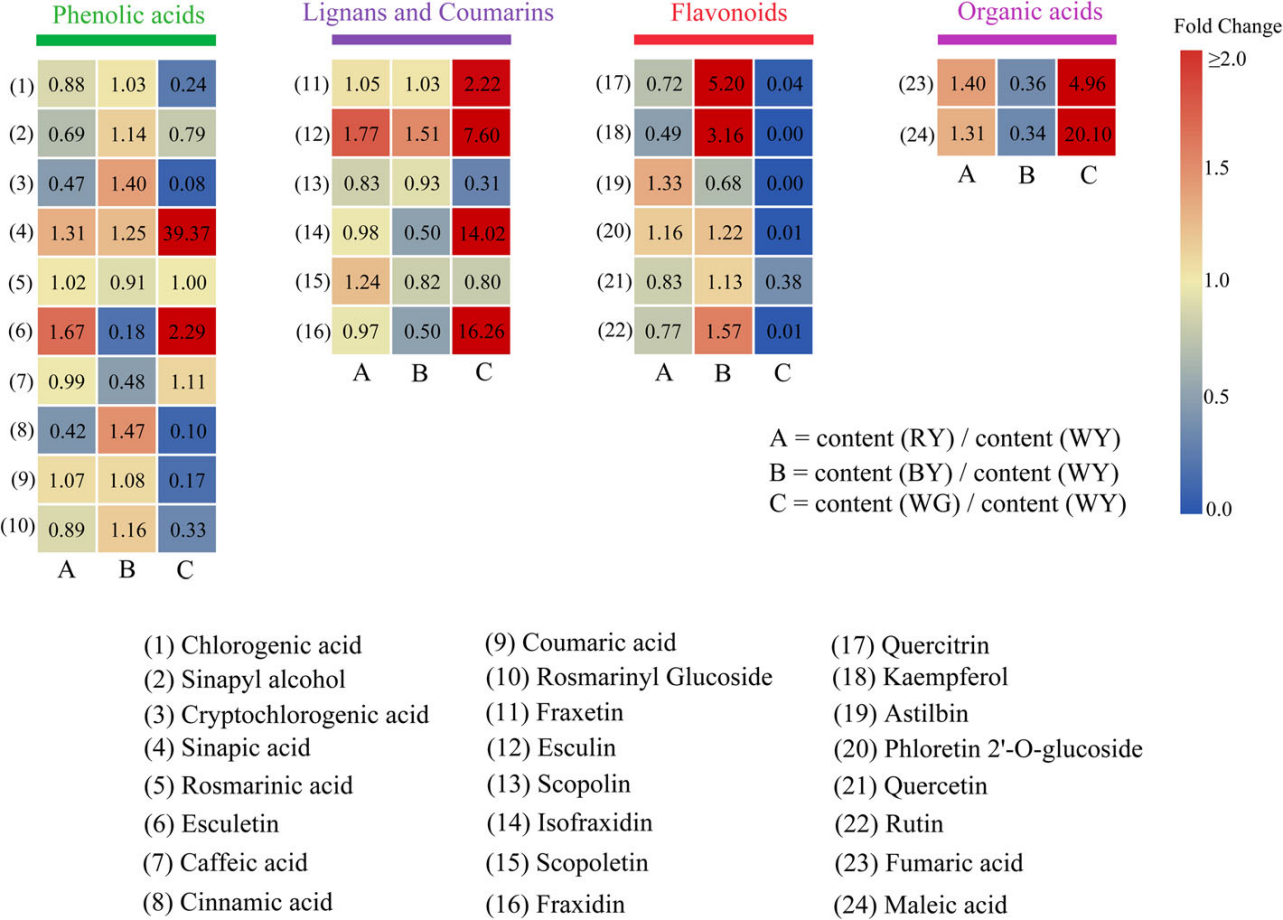
Heat map diagram of the relative concentration of the 24 principle active metabolites. In the upper legend, different colors represent different metabolite classes. The metabolite names are listed at the bottom. A: the fold change of relative content between RY and WY; B: the fold change of relative content between BY and WY; C: the fold change of relative content between WG and WY. Fold change ≥2 or ≤ 0.5, they were regarded as the differential metabolites

Furthermore, we selected the main active constituents and compared their accumulation patterns in WY, RY, BY, and WG tissues (Fig. 4). Relative to WY, the production of esculetin, caffeic acid, isofraxidin, fraxidin, fumaric acid, and maleic acid were significantly reduced (fold change ≤0.5), while the yields of quercitrin and kaempferol were significantly up-regulated in BY (fold change ≥2). Besides, the production of cryptochlorogenic acid, cinnamic acid, esculin, and rutin were increased by 1.5-fold in BY. Meanwhile, compared to WY, the production of sinapic acid, esculetin, esculin, astilbin, fumaric acid, and maleic acid were increased by 1.3- to 1.7-fold, whereas the contents of cryptochlorogenic acid, cinnamic acid, and kaempferol were significantly decreased in RY (fold change ≤0.5). Finally, compared to WY, the production of chlorogenic acid, cryptochlorogenic acid, cinnamic acid, coumaric acid, rosmarinyl glucoside, scopolin, quercitrin, kaempferol, astilbin, phloretin 2′-O-glucoside, quercetin, and rutin were significantly reduced (fold change ≤0.5), whereas the yields of sinapic acid, esculetin, fraxetin, esculin, isofraxidin, fraxidin, fumaric acid, and maleic acid were significantly promoted in WG (fold change ≥2).

### Candidate genes involved in the phenylpropanoid biosynthesis

**Fig. 5:**
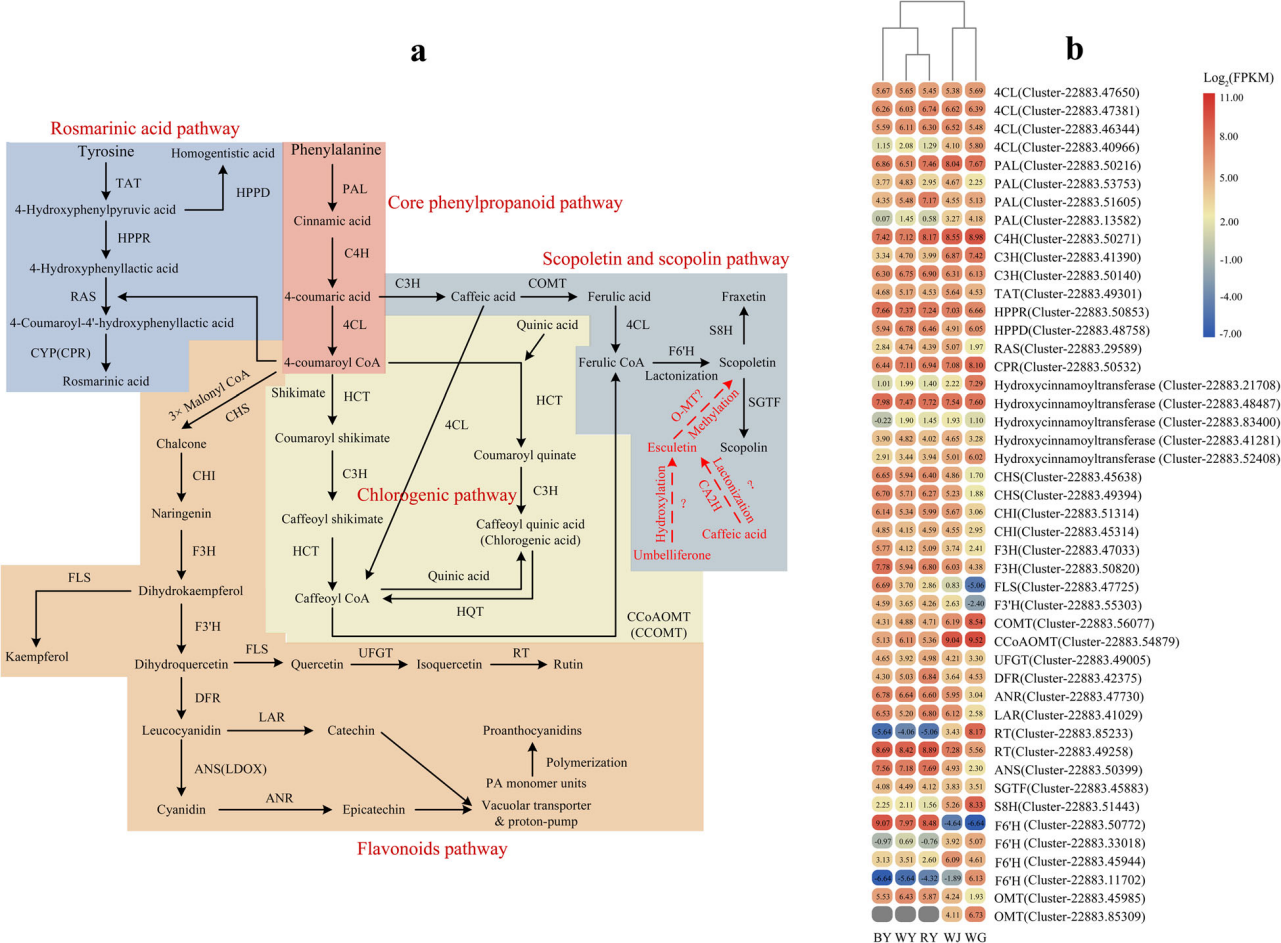
Phenylalanine metabolic pathways and heat map diagram of candidate genes. **a** Solid lines with arrow indicate that the enzymatic reactions are validated in previous studies, whereas dotted lines with arrow indicate that some steps of enzymatic reactions are not completely confirmed. **b** The name of genes are listed on the vertical line, and the samples are listed horizontally. The scale represents the logarithms of the FPKM values of these candidate genes. The grey rectangles without values mean that the FPKM values are zero

We have identified 46 candidate genes encoding most of the enzymes involved in the flavonoids, coumarins, and phenolic acid biosynthesis (Table 3, Fig. 5a, b, Additional file 8: Table S5, and Additional file 9: Table S6). Moreover, five genes related to rosmarinic acid biosynthesis were also found. The biosynthesis of flavonoids, phenolic acids, and most of the coumarins is derived from the core phenylpropanoid pathway, which is composed of three committed steps catalyzed by PAL, C4H, and 4CL enzymes. In this study, four *PAL* genes, four *4CL* genes, and one *C4H* gene were identified, of which the *PAL* gene (Cluster-22,883.50216), *4CL* gene (Cluster-22,883.47381), *C4H* gene (Cluster-22,883.50271) exhibited the highest expression levels compared to other members and showed higher expression levels in BY and RY. In the chlorogenic acid pathway, five genes encoding hydroxycinnamoyltransferases were found, and the expression level of gene (Cluster-22,883.48487) was much higher than other members. Besides, the gene (Cluster-22,883.48487) was highly expressed in BY. Furthermore, the gene (Cluster-22,883.21708) was only highly expressed in WG compared to other samples. The scopoletion biosynthesis is derived from a key precursor, namely ferulic CoA, produced by the enzymatic reactions of 3-O-methyltransferase (COMT) and 4CL on caffeic acid or by Caffeoyl CoA 3-O-methyltransferase (CCoAOMT) reacting with caffeoyl CoA. Compared to WY, only one *COMT* gene (Cluster-22,883.56077) and one *CCoAOMT* gene (Cluster-22,883.54879) were identified in this pathway, both of which have the highest expression levels in WG and down-regulated in RY and BY. Furthermore, five genes encoding 2-oxoglutarate and Fe (II)-dependent dioxygenases family proteins were characterized. Particularly, the F6’H and S8H proteins belong to 2-oxoglutarate and Fe (II)-dependent dioxygenases family*.* The unrooted phylogenetic tree showed that the proteins encoded by these five genes were highly similar to F6’H or S8H proteins (Additional file 10: Fig. S4). Among them, the protein encoded by a gene (Cluster-22,883.51443) from *S. glabra* showed maximum similarity with S8H protein (XP_030962790.1) from *Quercus lobata*. In addition, among these genes, the gene (Cluster-22,883.51443) was highly expressed in WG, while another gene (Cluster-22,883.50772) had a significantly higher expression level in BY compared to other samples. The pathway to the product of rosmarinic acid consists of four consecutive enzyme reactions, starting from the precursor (tyrosine) and four enzymes tyrosine aminotransferase (TAT), hydroxyphenylpyruvate reductase (HPPR), rosmarinate synthase (RAS), and cytochrome P450 reductase (CYP). These were encoded by genes of Cluster-22,883.49301, Cluster-22,883.50853, and Cluster-22,883.50532, respectively. For the rosmarinate synthase (RAS) protein, the unrooted phylogenetic tree showed that RAS protein encoded by the gene (Cluster-22,883.29589) from *S. glabra* had the maximum similarity with RAS proteins from *Juglans regia* (XP_018826314.1) and *Arachis ipaensis* (XP_016205435.1) Additional file 11: Fig. S5). The gene of Cluster-22,883.49301 showed no significantly differential expression level in WY, RY, and BY, but Cluster-22,883.50853 was highly expressed in BY. The gene of Cluster-22,883.50532 had a significantly higher expression level in WG, whereas its expression level was less in BY, relative to WY and RY.

**Table 3:** Summary of identified genes in the transcriptome of *S. glabra*

Gene	Number of genes	Gene	Number of genes	Gene	Number of genes
*PAL*	4	*CHS*	2	*ANR*	1
*4CL*	4	*CHI*	2	*ANS*	1
*C4H*	1	*F3H*	2	*TAT*	1
*C3H*	2	*F3’H*	1	*HPPR*	1
*COMT*	1	*FLS*	1	*HPPD*	1
*CCoAOMT*	1	*UFGT*	1	*RAS*	1
*F6’H*	4	*RT*	2	*CPR*	1
*S8H*	1	*DFR*	1	*Hydroxycinnamoyl-transferase*	5
*SGTF*	1	*LAR*	1	*O-MT*	2

The flavonoids biosynthesis shared the core pathway incorporating CHS, CHI, and F3H enzymes and the expression levels of these genes encoding the above-mentioned enzymes were higher both in RY and BY. As the intermediate compound (dihydrokaempferol) was produced by the core enzymatic reactions, it would be flowed into several branches to generate anthocyanins, proanthocyanidins, flavonols, etc. The biosynthesis of kaempferol is catalyzed by flavonol synthase (FLS) enzyme on dihydrokaempferol, and FLS enzyme was encoded by the gene of Cluster-22,883.47725, the expression level of Cluster-22,883.47725 was significantly up-regulated in BY. Rutin also originates from the precursor of dihydrokaempferol through the enzymatic reactions by flavonoid 3′-hydroxylase (F3’H, encoded by Cluster-22,883.55303), FLS, UDPG flavonoid O-glucosyltransferase (UFGT, encoded by Cluster-22,883.49005), and rhamnosyl transferase (RT, encoded by Cluster-22,883.85233 or Cluster-22,883.49258). These genes (except Cluster-22,883.85233) had similar expression patterns that BL had the potential to induce higher expression levels. Interestingly, the gene of Cluster-22,883.85233 was mainly expressed in WG to synthesize rutin, but Cluster-22,883.49258 showed a higher expression level in WY and WJ. A series of enzymes, including dihydroflavonol 4-reductase (DFR), leucoanthocyanidin reductase (LAR), anthocyanin synthase (ANS), and anthocyanidin reductase (ANR), are responsible for the production of catechin and epicatechin, which eventually synthesize the product of proanthocyanidins (PAs). The genes of *DFR* (Cluster-22,883.42375) and *LAR* (Cluster-22,883.41029) had higher expression levels in RY, while the gene of Cluster-22,883.47730 encoding ANR had a higher expression level in BY. Additionally, the gene of *ANS* (Cluster-22,883.50399) was highly expressed in RY and BY. Overall, the BL and RL cultivation increased the expression levels of many functional genes, such as some isoforms in the *PAL* gene family, *4CL* gene family, *CHS*, *CHI*, *F3H*, *C4H*, and *ANS*.

### Candidate transcription factors of MYB and bHLH

**Fig. 6:**
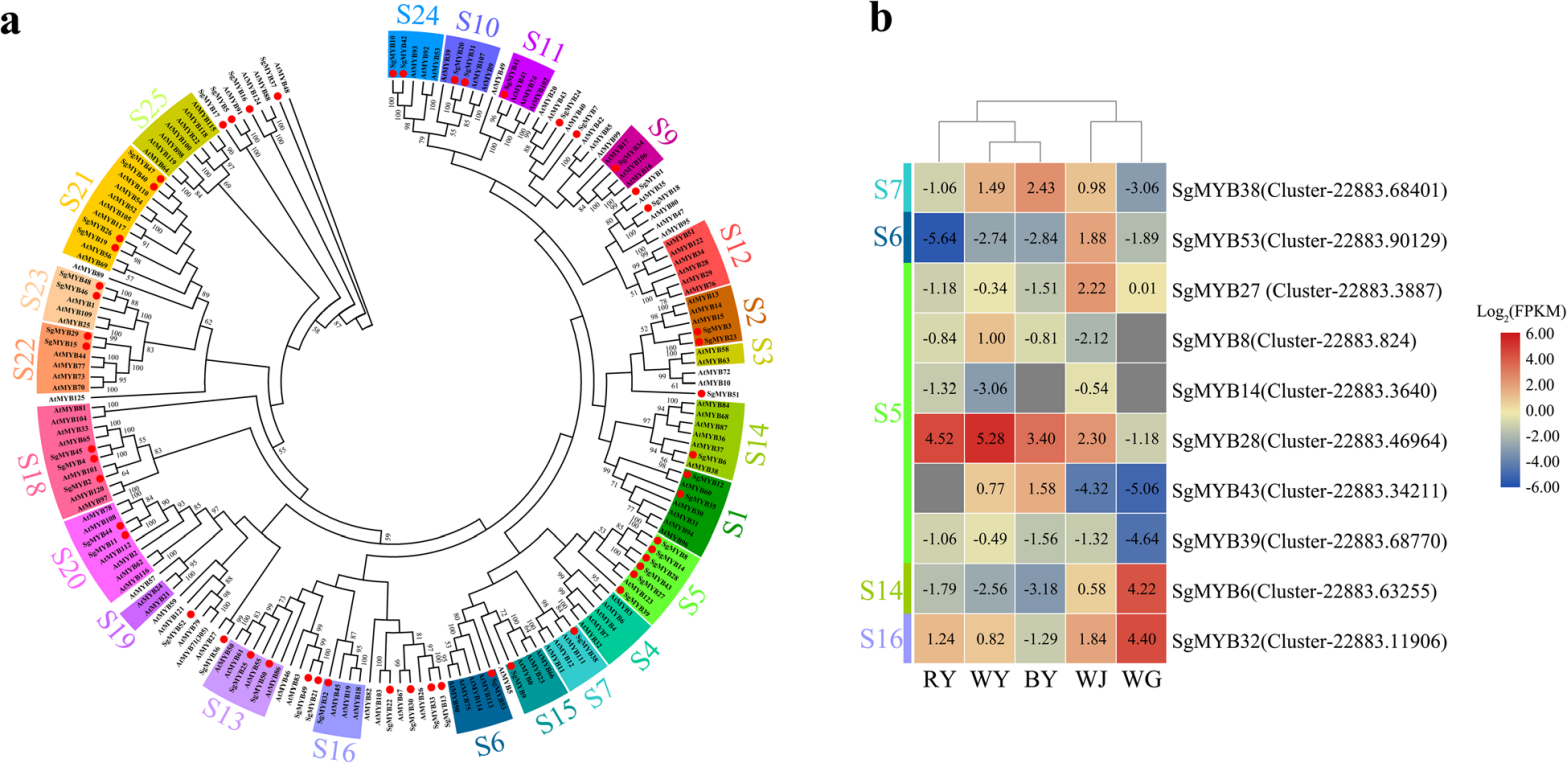
Phylogenetic tree and heat map diagram with R2R3-MYB proteins. Bootstrap values are displayed as percentages (1000 replicates) when > 50%; **a** The subgroups (S1-S25) of R2R3-MYB proteins in *Arabidopsis* highlighted with different colors were designated as previously reported [31, 35], and 53 genes encoding R2R3-MYB proteins of *S. glabra* were numbered randomly from 1 to 53. All the labeled R2R3-MYB amino acid sequences are given in Additional file 14: Table S7; **b** All the selected *R2R3-MYB* genes are listed on the vertical line. The scale represents the logarithms of the FPKM values of these genes. The grey squares without values mean that the FPKM values are zero

A total of 142 raw MYB (MYB and MYB-related) genes and 61 raw bHLH genes were collected. For MYB proteins, after screening, 92 MYB genes had different numbers of highly conserved DNA-binding domains and were divided into 4 classes: 1R-MYB, R2R3-MYB, 3R-MYB, and 4R-MYB proteins including 35, 53, 3, and 1 genes, respectively. Since the *Arabidopsis* genome was published, it has provided the first insight into the description and classification of plant MYB TFs. In the plants, R2R3-MYB proteins are the largest class in MYB family. They are involved in plant-specific processes and have been divided into subgroups in *Arabidopsis* based on the conservation of the DNA-binding domain and of amino acid motifs in the C terminal domain [31]. Here, we have identified all the primary structures of 53 R2R3-MYB domains in *S. glabra* (Additional file 12: Fig. S6)*.* We constructed the phylogenetic tree with these 53 R2R3-MYB amino acid sequences from *S. glabra* and 125 full-length R2R3-MYB amino acid sequences from *Arabidopsis thaliana* (Fig. 6a). Numerous studies have shown that some subgroups of R2R3-MYB proteins have been involved in the regulation of flavonoids biosynthesis in *Arabidopsis thaliana*, including subgroups of S4, S5, S6, and S7. In subgroup 7, AtMYB11, AtMYB12, and AtMYB111 were closely related to flavonols biosynthesis. SgMYB38 was clustered with AtMYB111. SgMYB53 showed a high degree of functional similarity within the subgroup of S6 (AtMYB75/90/113/114), regulating the anthocyanin pigments pathway. Six R2R3-MYB proteins of SgMYB8/14/28/43/27/39 were in the same branch of the PAs biosynthetic pathway with AtMYB123 in subgroup 5. Moreover, we identified two R2R3-MYB proteins: SgMYB6 with a high amino acid sequence similarity to AtMYB38 (RAX2, S14), and SgMYB32 clustered with members of subgroup 16. The heat map showed the expression patterns of selected 10 genes mentioned earlier according to the Log_2_(FPKM) values (Fig. 6b). Based on the transcriptome results, the expression level of *SgMYB38* (Cluster-22,883.68401) was up-regulated in BY. In subgroup 5, the six genes had different expression patterns among different samples. The genes of *SgMYB28* (Cluster-22,883.46964), *SgMYB8* (Cluster-22,883.824), and *SgMYB39* (Cluster-22,883.68770) exhibited the highest expression levels in WY, compared to RY and BY. Meanwhile, the expression level of *SgMYB43* (Cluster-22,883.34211) displayed a significant increase in BY. Remarkably, the gene of *SgMYB27* (Cluster-22,883.3887) presented the highest expression level in WJ, while the genes of *SgMYB6* (Cluster-22,883.63255) and *SgMYB32* (Cluster-22,883.11906) were highly expressed in WG.

**Fig. 7:**
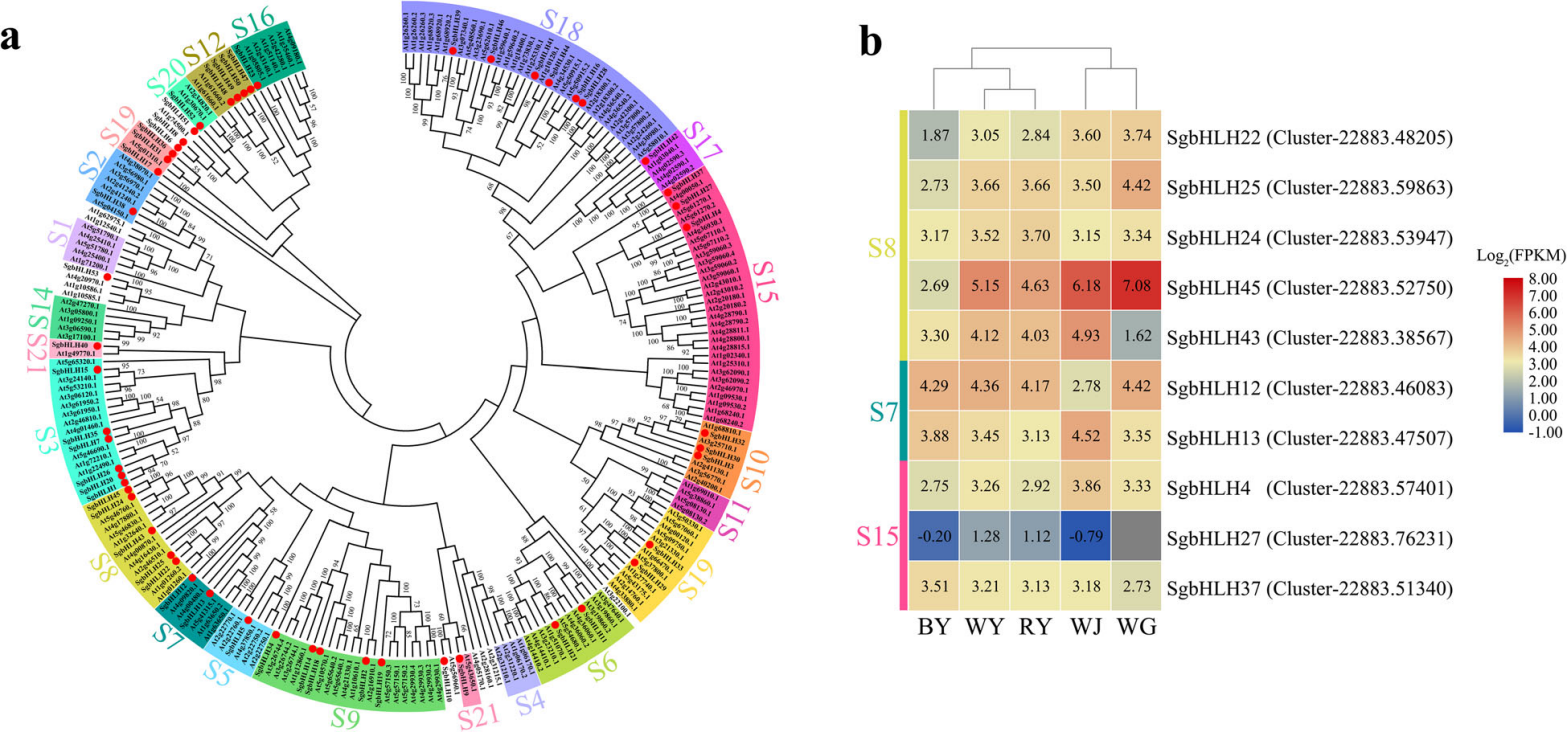
Phylogenetic tree and heat map diagram with bHLH proteins. Bootstrap values are displayed as percentages (1000 replicates) when > 50%; **a** The subgroups S1-S21 for bHLH proteins in *Arabidopsis* highlighted with different colors were designated as previously reported [33], and 53 genes encoding bHLH proteins of *S. glabra* were numbered randomly from 1 to 53. All the labeled bHLH amino acid sequences are given in Additional file 13: Table S6; **b** All the selected *bHLH* genes are listed on the vertical line. The scale represents the logarithms of the FPKM values of these genes. The grey squares without values mean that the FPKM values are zero

The bHLH proteins are another ancient and functionally diverse superfamily of TFs, which have been widely investigated in the past. In this study, we identified 53 genes encoding bHLH proteins that had the distinguishable signature domain. We constructed the phylogenetic tree with these 53 bHLH amino acid sequences from *S. glabra* and 182 full-length AtbHLH protein sequences from *Arabidopsis thaliana* (Fig. 7a). The results of multiple sequences alignment of SgbHLH domains have been displayed in Additional file 13: Fig. S7. As shown in Fig. 7a, all the bHLH proteins were divided into 21 subclasses indicating that the node values between different subclasses were low, but values within each subclass were high, suggesting that the latter have strong evolutionary relationships. Particularly, a previous study showed that the bHLH proteins were classified into 32 subclasses [36], with subclasses of S2, S5, and S24 (corresponding to subclasses of S8, S7, and S15 of AtbHLH proteins) involved in the regulation of flavonoids biosynthesis. In the subclass of S8, we identified a group of SgbHLH proteins, namely SgbHLH22/24/25/43/45, which showed high sequence similarity with other AtbHLH protein members. Compared to the similar gene-expression change between the WY and RY, the relative expression levels of these five genes encoding SgbHLH proteins from S8 were significantly lower in BY. Particularly, the gene of *SgbHLH45* (Cluster-22,883.52750) was significantly up-regulated in WJ and WG. Within the subclass of S7, SgbHLH13 and SgbHLH12 were clustered with the subset of At1g63650 (EGL), At5g41315 (GL3), At4g00480 (MYC1), and At4g09820 (TT8) that associated with some R2R3-MYB members to participate in the regulation of flavonoids metabolism, trichome formation, epidermal cell fate specification, and in the formation of hair and non-hair cells in root epidermis cells [36]. The gene of *SgbHLH12* (Cluster-22,883.46083) showed no significantly differential expression pattern in WY, RY, BY, and WG, but it had a lower expression level in WJ. However, the gene of *SgbHLH13* (Cluster-22,883.47507) had the highest expression level in WJ. Furthermore, three bHLH proteins of SgbHLH4/27/37 belonged to the subclass of S15. The expression level of *SgbHLH4* (Cluster-22,883.57401) was slightly reduced in BY and RY, compared to WY, but *SgbHLH27* (Cluster-22,883.76231) showed a lower level in BY, WJ, and WG. The gene of *SgbHLH37* (Cluster-22,883.51340) had a much higher expression level in BY (Fig. 7b).

### Validation of the transcript expression using qRT-PCR

**Fig. 8:**
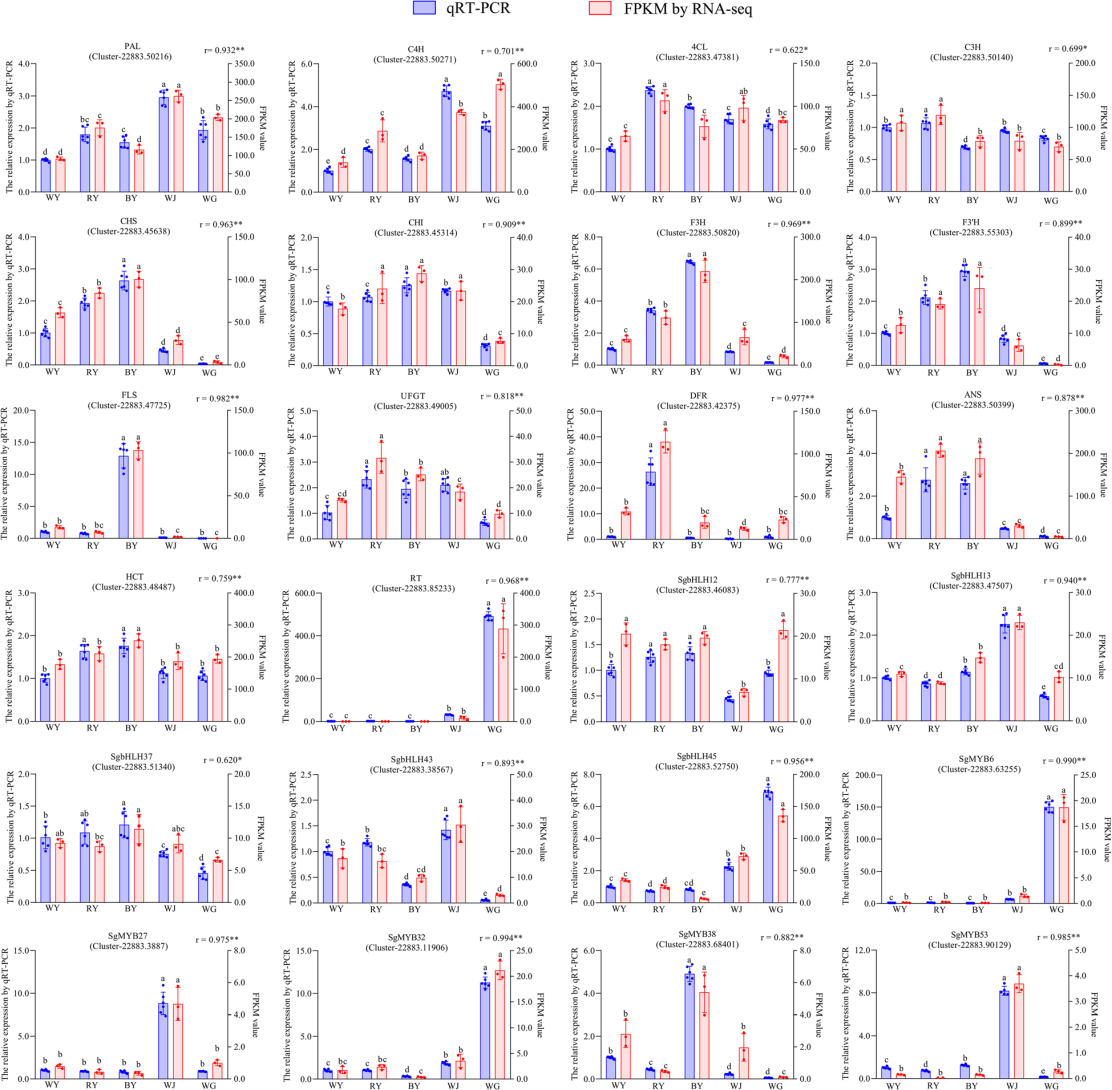
qRT-PCR validation of the 24 selected genes. The left y-axis and white legend represent the relative expression levels determined by qRT-PCR, while the right-axis and slash legend represent the FPKM values obtained by RNA-seq. Expression values were adjusted by setting the expression of WY to 1 for each gene. All qRT-PCRs for each gene used triplicates with two repeats per experiment. Error bars indicate SD, and different lower case letters (a-e) represent a significant difference among the five samples at *P*-value < 0.05. All the individual points were plotted in the bar plots. The correlation coefficient (r) between qRT-PCR and RNA-seq for each gene was shown with its corresponding significance level (** *P* < 0.01, * *P* < 0.05)

To confirm the accuracy of the RNA-seq results, a total of 24 genes (five R2R3-MYB genes, five bHLH genes, and another 14 functional genes related to flavonoids biosynthesis) were selected (Additional file 8: Table S5). The qRT-PCR results revealed that the expression levels of 24 selected genes were mostly consistent with those obtained by RNA-seq (Fig. 8). Furthermore, for each selected gene, the high correlation coefficient (r > 0.600) was found between qRT-PCR and RNA-seq. Consequently, the findings of the qRT-PCR revealed that the RNA-seq data were reliable and could be used in future experiments.

## Discussion

### Effects of different LED lights on plant morphological traits

In this study, we found that monochromatic LED lights had a significant influence on the morphological characteristics of *S. glabra*. Specifically, compared to WL, the RL significantly increased the plant height and decreased the stem diameter and leaf area, whereas BL significantly suppressed both the height and leaf area of *S. glabra*. Similar findings were also obtained in previous studies, for instance, radish stems were found to be thin and elongated under RL [37]. In experiments on the effects of BL and RL on stem elongation of tomato seedlings and other species, results showed that stem length decreased by increasing BL to RL ratio [19, 38]. However, another report on cucumber showed that seedlings showed increased height, hypocotyl, and epicotyl under BL compared to other treatments [39]. Red and blue light are both significant components of the spectrum of photosynthetically active radiation (PAR) and hence play a fundamental role in plant photomorphogenesis. Plants sense and transmit light signals through photoreceptors such as phytochromes (Phy) [36, 40], cryptochromes (Cry) [41] and phototropins (Phot) [42], which in turn trigger plant growth and development by signal transduction. Phytochromes are R/FR light photoreceptors that can constantly perceive and respond to the surrounding environment [43]. Under irradiation by RL, phytochromes suppress the activity of CONSTITUTIVE PHOTOMORPHOGENCI 1 (COP1) and PHYTOCHROME INTERACTING FACTORs (PIFs) proteins. The expression level of the gene (Cluster-22,883.48222) encoding the COP1 protein was significantly reduced in RY compared to WY and BY, implying that RL accelerated Phys to break down COP1 and thus induced several photomorphogenesis-promoting TFs (e.g., HY5 and LAF1), as evidenced by their higher expression level [40]. In this study, we found that the genes of Cluster-22,883.57986 encoding HY5 and *SgMYB32* (Cluster-22,883.11906) encoding LAF1 showed higher expression levels to various degrees.

The Cry family members, Cry1, Cry2, and Cry3 (Cry-DASH), which primarily mediate BL and involve in the inhibition of hypocotyl elongation, de-etiolation of seedlings, floral initiation, root development, and other photoresponses in *Arabidopsis thaliana* [41], whereas the Cry family members are diverse among different plants [38]. Meanwhile, the Phot family members (Phot1 and Phot2) are recognized as another Ultra-violet (UV) and blue-light photoreceptors. Phot1 and Phot2 are involved in the regulation of phototropism in different regulatory patterns: Phot1 activation needs a low threshold of BL intensity, while Phot1 and Phot2 are mediated coherently by higher blue light intensities [38, 42]. In terms of hypocotyl elongation, previous research demonstrated that its inhibition was controlled by the combined action of Cry1, Cry2, and Phot1 during the de-etiolation stage of young seedlings [38, 41]. In this study, we found that four genes of Cluster-22,883.51204, Cluster-22,883.48125, Cluster-22,883.53309, and Cluster-22,883.50402 putatively encoding Phot1, Phot2, Cry1, and Cry2 proteins, respectively, and they were involved in the perception and response to blue light irradiation (Additional file 15: Fig. S8). Among these genes, the relative expression levels of *Phot1* (Cluster-22,883.51204), and *Cry1* (Cluster-22,883.53309) genes were up-regulated in BY, however, the *Cry2* (Cluster-22,883.50402) gene was down-regulated. As mentioned earlier, the higher expression level of *Phot1* and *Cry1* genes inhibited hypocotyl elongation in *S. glabra* seedlings, which were subsequently integrated with other signaling molecules to mediate the regulatory network of those blue light-regulated genes by transcriptional and post-translational mechanisms [41, 44]. In a previous study using a kinetic analysis method, it was reported that the rate of stem growth was determined by multiple environmental signals, such as hormones and endogenous rhythms [45]. The explanation of why different evolutionary phenotypic and aerial development responses to RL, BL, and BL/ RL were inconsistent among different plants is complex and needs further study. Furthermore, the detailed mechanism of phytochrome-mediated regulation (e.g., stem elongation and bud outgrowth, also warrants further research [43].

### The conjoint analyses of the production of flavonoids and coumarins biosynthesis

All of the flavonoid compounds identified in this study are thought to be derived directly from the reaction between 4-coumaroyl CoA and 3 units of malonyl CoA catalyzed by CHS, CHI, F3’H, F3H, and FLS enzymes (encoded by early biosynthetic genes, EBGs). The intermediate products of this reaction are dihydroflavonols (e.g., dihydrokaempferol or dihydroquercetin) which are subsequently used as substrates in other pathways involved in flavonol, anthocyanin, and proanthocyanidin biosynthesis regulated by downstream genes (designated late biosynthetic genes (LBGs), Fig. 5a) [46–49]. Our analysis of metabolism profiling (Fig. 4) revealed that the yield of some flavonols (e.g., quercitrin and kaempferol) were significantly higher in BY. They play an important role in pollen fertility, flower color, and UV-B protection [50]. In flavonol metabolism, FLS, belonging to 2-oxoglutarate-dependant dioxygenase, is a branch-point enzyme with multiple isoforms that catalyzes the conversion from dihydrokaempferol to products in a tissue-specific and inducible manner [51, 52]. In this study, the gene (Cluster-22,883.47725) encoding FLS enzyme was identified. We noticed that flavonol production varied according to its relative expression in WY, RY, BY, and WG. Given the higher relative expression of *FLS* gene in the blue light treatment, the production of quercitrin, kaempferol, and rutin increased drastically in the leaves of *S. glabra.* Cinnamic acid and 4-coumaric acid are both upstream products of the first two steps of the core phenylpropanoid pathway, their production thus regulates the flow of precursors to all side branches in connection with the biosynthesis of lignans, flavonoids, and phenolic acids. Among these, lignins are synthesized by the conversion from 4-coumaric acid to caffeic acid catalyzed by C3H enzyme, and caffeic acid subsequently enters the lignins pathway. Meanwhile, the 4CL enzyme catalyzes the conversion of 4-coumaric acid to 4-coumaroyl CoA, a substrate assigned into the chlorogenic pathway in combination with shikimate or quinic acid, and it also proceeds to all classes of flavonoids production together with malonyl CoA. According to metabolite analysis, compared to WY, the initial products of cinnamic acid and 4-coumaric acid contents were increased by 1.47-fold and 1.08-fold in BY, respectively, but the bypass of caffeic acid production was inhibited in BY. Therefore, the increasing production of 4-coumaric acid led to more synthesis of 4-coumaroyl CoA, which in turn generated a higher production of flavonoids and chlorogenic (e.g., chalcone and naringenin). Generally, the accumulation of initial precursors is closely related to the expression level of biosynthetic genes, including *PAL*, *C4H*, and *4CL* genes that are the three consecutively committed enzymes in core phenylpropanoid pathway leading to varieties of flavonoids. In *A. thaliana*, the *PAL* gene family (*AtPAL1*-*AtPAL4*), *4CL* gene family (*At4CL1-At4CL4*), and only one member of *AtC4H* have been identified and elaborated, some of which-like *AtPAL1* and *AtPAL2 -*are thought to have a major role in the phenylpropanoids pathway [28, 53], while *At4CL3* is thought to has a preference for the flavonoids biosynthesis [54]. The unrooted phylogenetic tree showed that the genes of Cluster-22,883.50216, Cluster-22,883.50271, and Cluster-22,883.47381 were highly homologous with *PAL1/2*, *C4H*, and *4CL3* genes, respectively, which exhibited the up-regulated expression levels under blue and red light condition, suggesting that these consecutive genes served to the flavonoids biosynthesis (Additional file 16: Fig. S9).

Coumarins are widely distributed in numerous plant species and encompass multiple bioactivities in defense against phytopathogens, abiotic stress, oxidative stress, clinical diseases, and are the crucially specialized metabolites of *S. glabra* [4, 5]. Due to the crucial enzymatic steps related to coumarins biosynthesis are still largely unknown in plants including *S. glabra*, this study investigated the current possible pathways and analyzed the genes encoding F6’H, S8H, and scopoletin glucosyltransferase (SGTF) involved in the biosynthesis of scopoletin, fraxetin, and scopolin. In Fig. 4, except scopolin and scopoletin, the productions of fraxetin, esculin, isofraxidin, and fraxidin were most abundant in WG. Regrettably, the isofraxidin and fraxidin biosynthesis are not mapped in KEGG pathway database. The substantial accumulation of fraxetin was closely related to the higher expression level of the gene (Cluster-22,883.51443) encoding S8H in WG. Furthermore, the yield of scopolin was decreased in RY, BY, and WG because of the lower expression level of *SGTF* gene (Cluster-22,883.45883), which catalyzes scopolin formation. As for scopoletin, apart from the points on the formation of scopoletin derived from ferulic acid by enzymatic reaction of 4CL and F6’H1 in turn [55]. Other studies revealed that scopoletin could also be produced from the conversion of esculetin via the methylation of O-methyltransferase (O-MT) [4]. Under the BL condition, the reduced production of scopoletin might be caused by the less yields of its both precursors of ferulic acid and esculetin. Similar to scopoletin, the biosynthetic pathway of esculetin originated from umbelliferone or caffeic acid on earth that is still unclear [4].

### Some R2R3-MYB and bHLH TFs regulate the expression levels of EBGs and LBGs

To date, there have been various studies on TFs of R2R3-MYB, bHLH, WDR, and MBW complexes as regulators of the phenylpropanoid biosynthesis in plants [48, 56–60]. These studies highlighted the importance of these TFs and MBW complexes in the regulation of flavonoids biosynthesis in plant tissues and the seed coat. The complete flavonoids pathway relies on the coordinated expression of EBGs and LBGs. Moreover, in *A. thaliana*, the subclasses (S4, S5, S6, and S7) of R2R3-MYB proteins [31], the subgroups (S8, S7, and S15) of bHLH proteins [33], and several WD40 proteins [34, 61] (e.g., TTG1) have been proved to be involved in the regulation of the expression of EBGs and LBGs in the flavonoids biosynthesis in a certain way. In flavonol biosynthesis, the EBG genes are regulated by members of AtMYB11/12/111 proteins from subgroup 7, and consequently control the development of flavonol [47, 62]. SgMYB38 was assigned to subgroup 7 and was likely to control the expression level of *CHS*, *CHI*, *F3H*, *F3’H*, and *FLS* genes. Compared to WY and RY, *SgMYB38* gene was significantly up-regulated in BY, which directly increased the expression levels of EBGs and promoted the accumulation of some flavonol compounds. In addition, the results of qRT-PCR and RNA-seq data of these EBGs have demonstrated it. The accumulation of PAs is mainly distributed in the innermost cell layer of the seed coat regulated by more than four MBW complexes, i.e., AtMYB123 (TT2)-TT8/GL3/EGL3-TTG1, and AtMYB5-TT8-TTG1, which target the different LBGs, such as *ANS (LDOX)*, *DFR*, and *UFGT* [30]. The MBW complexes of AtMYB75/90/113/114-TT8/GL3/EGL3-TTG1 also determine anthocyanin biosynthesis via the regulation of the spatial and temporal expression of LBGs in vegetative tissues [63]. Based on these results, it is reasonable to assume that MBW complexes target gene specificity. Therefore, in this study, we screened and identified some genes encoding R2R3-MYB, bHLH and WDR TFs. Among these TFs, SgMYB8/14/27/28/43/39 might encode the homologous protein TT2 belonging to subgroup 5 of R2R3-MYB proteins, while the protein encoded by *SgMYB53* showed a high degree of functional similarity within members in subgroup 6 of R2R3-MYB proteins. SgbHLH12/13 were predicted to encode TT8/GL3/EGL3/MYC1 belonging to subgroup 7 of bHLH proteins in *Arabidopsis*, while the gene of Cluster-22,883.52183 was largely thought to encode TTG1. It is reasonable to assume that the assembled MBW complexes of SgMYB8/14/27/28/43/39-SgbHLH12/13-TTG1 might be involved in the synthesis of PAs, while SgMYB53-SgbHLH12/13-TTG1 were likely to be associated with anthocyanins biosynthesis. In this study, we just identified these genes encoding TFs and proposed potential MBW complexes in *S. glabra* according to the studied model plants. However, some aspects of the flavonoid biosynthesis still remain unknown: for instance, due to the unavailable genomic data for *S. glabra*, the copy numbers of important genes involved in the phenylpropanoid pathway have not yet been identified. An in-depth analysis of the MBW complexes structure, function, and regulation in *S. glabra* is still required.

## Conclusions

In conclusion, *S. glabra* seedlings cultured under different monochromatic LED lights (WL, RL, and BL) showed significant differences in plant growth. Additionally, a combined analysis of transcriptomic and metabolomic profiling demonstrated that LED lights had a significant influence on metabolites accumulation and the expression patterns of functional genes and TFs (R2R3-MYB and bHLH) involving in phenylpropanoid biosynthesis. Compared to WL, BL showed higher expression levels in EBGs and some LBGs under the regulation of specific TFs, leading to a significant increase in flavonoid production in BY. *FLS* gene acted as a limiting factor, reducing the yield of flavonol compounds in the RL condition. Furthermore, the production of desired compounds (e.g., esculetin, isofraxidin, fraxidin, fumaric acid, and maleic acid) was significantly reduced in BY, while RL stimulated the accumulation of some vital compounds (e.g., sinapic acid, esculetin, esculin, scopoletin, astilbin, fumaric acid, and maleic acid) by 1.24- to 1.77-fold in RY. This research offers useful insights into the regulatory mechanism of the phenylpropanoid biosynthesis in *Sarcandra glabra*.

## Methods

### Treatments and samples collection

Seeds of *S. glabra* were collected from the Louyuan state-owned forest farm in Sanming City, Fujian Province, China. The seeds were then cultivated at constant temperature and humidity incubator (25 °C, and 60% humidity) in the Bamboo Institute of the Fujian Agriculture and Forestry University, Fuzhou, Fujian Province, China (26°5′ N, 119°13′ E). When the first pair of true leaves were fully expanded, the 90 seedlings were transplanted into black Poly Vinyl Chloride (PVC) rectangular pots (10 × 10 × 8 cm) using Pindstrup substrate (pH 5.5) for cultivation. Seedlings were arranged randomly in the culture room under three different light treatments (WL, 380 ~ 760 nm; RL, 656 nm; BL, 450 nm). Each treatment (WL, RL, and BL) had 30 seedlings, making 90 seedlings in total, and in each treatment (30 seedlings), we further divided into 10 seedlings in the same treatments. The LED lights, quantum sensor, and light meter (HR-450) were purchased from Hipoint Corporation. The photosynthetic photon flux density (PPFD) of the lights was set as 80 μmol·m^− 2^·s^− 1^, and the photoperiod was 16/8 h (day/night). The respective intensities of the PPFD and light spectra were monitored using a HR-450 machine. After 60 days, we selected nine healthy seedlings from each treatment that were pulled out from substrates carefully, washed, and dried on filter paper. In each treatment, among those nine seedlings, three seedlings were selected to make a composite sample (one replicate). Thereafter, leaves, stems, and roots from each replicate were separated and the same tissues in each treatment were mixed. In this study, we primarily focused on the effects of WL, RL, and BL on leaves, as well as the influence of WL on different tissues *S. glabra*. Therefore samples of WY, RY, BY, WJ, and WG were selected with three replication and immediately frozen in liquid nitrogen and kept at-80 °C until further analysis.

### Determination of morphological traits

Plant height and stem diameter were measured by using a measuring tape and a vernier caliper, respectively throughout the cultivation period. After 60 days, the upper fully expanded leaves were measured using a portable leaf area meter (LI-3000C, Ecotek).

### RNA extraction and transcriptomic profiling

Total RNA was extracted from the samples using RNAprep Pure Plant Kit (DP441, TIANGEN BIOTECH). These RNA samples were then treated with DNase I (Takara) to remove genomic DNA contamination and sent to the Novogene Bioinformatics Technology Company (Beijing, China) for cDNA libraries construction and Illumina sequencing. The cDNA libraries were generated using NEBNext Ultra™ RNA Library Prep Kit for Illumina (NEB, USA) following the manufacturer’s recommendations. The library preparations were sequenced on an Illumina Hiseq platform and paired-end reads were generated.

### De novo assembly and annotation

For the quality of assembly, clean reads were obtained by removing reads containing adapter, reads containing ploy-N and low quality reads from raw data. The Trinity method was employed to de novo assemble the clean reads [64]. Seven public databases or programs were used to annotate the genes, including the NCBI non-redundant protein (Nr), the NCBI nucleotide sequences (Nt), the protein family (Pfam), the euKaryotic Ortholog Groups (KOG), the Swiss-Prot database, the Kyoto Encyclopedia of Genes and Genomes (KEGG) and the Gene Ontology (GO).

### Gene expression analysis

The fragments per kilobase of transcript per million mapped transcript (FPKM) values were used to analyze gene expression. The differential expression analysis of any two sets of treatments was measured using the R package *DEGseq2* [65]. An adjusted *P*-value of 0.05 was set as the threshold to determine significant differences in DEGs. The enrichment analysis was performed with GO ontology using the R package *GOseq* [66]. The KEGG pathway was annotated using the KEGG database. The corrected *P*-value < 0.05 and |log_2_(FoldChange)| ≥ 1 were set as the threshold to determine the significant differences under the GO and KEGG enrichment analyzes. Lastly, the heat map diagrams of the selected genes were constructed using TBtools software with the Log_2_ conversion of FPKM values [67].

### Candidate transcription factors of MYB and bHLH

According to the transcriptome data of *S. glabra*, we used the PlnTFDB database (http://planttfdb.cbi.pku.edu.cn/) to identify MYB and bHLH TFs [68]. Based on previous studies by Dubos and Stracke who have identified the characteristics and classification of the *R2R3-MYB* gene family in *Arabidopsis thaliana* [31, 35], we identified and classified the genes encoding R2R3-type MYB proteins in *S. glabra.* In addition, as per previous studies [33, 69] on the characteristics of the bHLH proteins in *Arabidopsis thaliana*, we identified the distribution and predicted DNA binding features of the bHLH proteins in *S. glabra*.

### Validation of RNA-seq data by qRT-PCR

First-strand cDNA was generated from 1 μg of total RNA using the PrimeScript™ RT reagent Kit with gDNA Eraser (RR047A, Takara, Japan). The gene-specific primers and a reference gene (clathrin adaptor complexes, CAC) were designed by Primer Premier 5.0 software (Primer, Canada), and they were synthesized by the SunYa Company (Fuzhou, China) (Additional file 17: Table S8). All analyses were repeated twice for each biological replicate. The qRT-PCR was performed on the ABI 7500 Real-Time PCR system (Applied Biosystems, USA) using SYBR Green premix Ex Taq Kit (RR820A, Takara, Japan). The expression levels of the tested reference genes were determined from C_T_ values and calculated using the 2^-ΔΔC^_T_method.

### Phylogenetic analysis

A multiple sequence alignment analysis was performed using the DNAman software. Phylogenetic trees were constructed by using MEGA7.0 with a neighbor-joining method and 1000 bootstrap replicates after alignment by ClustalW [70].

### Metabolomic profiling analysis

#### Sample preparation and extraction

Meanwhile, the samples of WY, RY, BY, and WG with three replication were sent to Metware Technology Company (Wuhan, China) for metabolomic profiling. The freeze-dried leaf and root were crushed using a mixer mill with a zirconia bead for 90 s at 30 Hz. One hundred milligram powder was weighted and extracted overnight at 4 °C using 0.6 ml 70% aqueous methanol. Following centrifugation at 10, 000×g for 10 min, the extracts were absorbed and filtrated before UPLC-MS/MS analysis.

#### UPLC conditions

The sample extracts were analyzed using a UPLC-ESI-MS/MS system (UPLC, Shim-pack UFLC SHIMADZU CBM30A system; MS, Applied Biosystems 4500Q TRAP). The analysis conditions were as follows, UPLC: column, Waters ACQUITY UPLC HSS T3 C18 (1.8 μm, 2.1 mm × 100 mm); The mobile phase consisted of solvent A, pure water with 0.04% acetic acid, and solvent B, acetonitrile with 0.04% acetic acid. Sample measurements were performed under a gradient program that employed the starting conditions of 95% A and 5% B. Within 10 min, a linear gradient to 5% A and 95% B was programmed, and a composition of 5% A, 95% B was maintained for 1 min. Subsequently, a composition of 95% A and 5.0% B was adjusted within 0.10 min and maintained for 2.9 min. The column oven was set to 40 °C, and the injection volume was 4 μl. The effluent was alternatively connected to an ESI-triple quadrupole-linear ion trap (QTRAP)-MS. A quality-control sample was made of an equal mixture of all samples. During the process, to monitor the stability of the analytical conditions, the quality-control sample was run after every ten injections.

#### ESI-Q TRAP-MS/MS

LIT and triple quadrupole (QQQ) scans were acquired on a triple quadrupole-linear ion trap mass spectrometer (Q TRAP), API 4500 Q TRAP UPLC/MS/MS System, equipped with an ESI Turbo Ion-Spray interface, operating in positive and negative ion mode and controlled by Analyst 1.6.3 software (AB Sciex). The ESI source operation parameters were as follows: an ion source, turbo spray; source temperature 550 °C; ion spray voltage (IS) 5500 V (positive ion mode)/− 4500 V (negative ion mode); ion source gas I (GSI), gas II (GSII), curtain gas (CUR) were set at 50, 60, and 30.0 psi, respectively; the collision gas (CAD) was high. Instrument tuning and mass calibration were performed with 10 and 100 μmol L^− 1^ polypropylene glycol solutions in QQQ and LIT modes, respectively. QQQ scans were acquired as MRM (multiple reaction monitoring) experiments with collision gas (nitrogen) set at 5 psi. DP and CE for individual MRM transitions were done with further optimization of the two parameters. Specific sets of MRM transitions were monitored for each period according to the metabolites eluted within the period.

### Metabolite identification and analysis

Metabolite identification was conducted by the self-compiled MWDB database (MetWare biological science and Technology Co., Ltd., Wuhan, China) and publicly available metabolite databases. Quantitative analysis of metabolites was done based on the MRM mode, and the characteristic ions of each metabolite were screened through the QQQ mass spectrometer to obtain signal strengths. Integration and correction of chromatographic peaks were performed using MultiQuant version 3.0.2 (AB SCIEX, Canada). The corresponding relative metabolite contents were shown as chromatographic peak area integrals. To investigate the metabolic differences and the degree of variation between samples and quality control sample (a mixer sample pooled together with equal mass from the aforementioned samples) with three replications, the unsupervised principal component analysis (PCA) was performed using the R package *prcomp*. To investigate metabolic changes in different samples, a hierarchical cluster analysis (HCA) was carried out using the R package *heatmap*, and the raw data was disposed of unit variance (UV) scaling before HCA analysis. The significantly differential metabolites were set with the thresholds of VIP (variable importance projection) ≥ 1 and Log_2_(Fold change) ≥ 1 or ≤ − 1.

### Statistical analysis

Statistical analyses were performed using the SPSS 20.0 software (IBM, USA). Differences in comparison of mean between different treatments were performed by analysis of variance (ANOVA) at α 0.05, 0.01, 0.001, and 0.0001 probability level. Where *P* < 0.05, *P* < 0.01, *P* < 0.001, and *P* < 0.0001 are denoted by *, **, ***, and ****, respectively. Graph-Pad Prism 8.0 and MS Excel were used for graphs and tables, respectively.

## Supplementary information

**Additional file 1: Table S1.** The statistics of leaf area, plant height, and stem diameter under different LED lights.

**Additional file 2: Table S2.** The statistics of the de novo assembly based on RNA-seq data.

**Additional file 3: Table S3.** The statistics of the annotated unigenes in seven databases.

**Additional file 4: Figure S1.** The histogram of GO annotation of all unigenes.

**Additional file 5: Figure S2.** Enriched GO Terms among BY vs. RY (Fig.S2a), BY vs. WY (Fig.S2b), WG vs. WY (Fig.S2c), WJ vs. WG (Fig.S2d), and WJ vs. WY. (Fig. S2e) groups.

**Additional file 6: Figure S3.** KEGG enrichment among BY vs. RY (Fig.S3a), BY vs. WY (Fig.S3b), RY vs. WY (Fig.S3c), WG vs. WY (Fig.S3d), WJ vs. WG (Fig.S3e), and WJ vs. WY (Fig.S3f) groups.

**Additional file 7: Table S4.** The statistics of the detected metabolites in *S. glabra.*

**Additional file 8: Table S5.** Summary of identified genes and their FPKM values.

**Additional file 9: Table S6.** The transcript sequences of the selected genes.

**Additional file 10: Figure S4.** Phylogenetic tree constructed on the basis of 19 amino acid sequences belonging to 2-oxoglutarate (2OG) and Fe (II)-dependent oxygenase superfamily proteins.

**Additional file 11: Figure S5.** Phylogenetic tree constructed on the basis of 24 amino acid sequences belonging to hydroxycinnamoyltransferases.

**Additional file 12: Figure S6.** The primary structure of 53 R2R3-MYB domains in *S. glabra*.

**Additional file 13: Figure S7.** Multiple sequence alignment of bHLH domains of the 53 members of the SgbHLH proteins family.

**Additional file 14: Table S7.** One hundred twenty-five full-length R2R3-MYB amino acid sequences from *Arabidopsis,* and 53 R2R3-MYB amino acid sequences from *S. glabra*; 182 full-length AtbHLH amino acid sequences from *Arabidopsis*, and 53 SgbHLH protein sequences from *S. glabra.*

**Additional file 15: Figure S8.** Phylogenetic tree constructed on the basis of 11 amino acid sequences belonging to Cryptochromes (Cry) family proteins (Fig.S8a); Phylogenetic tree constructed on the basis of 6 amino acid sequences belonging to Phototropin (Phot) family proteins (Fig. S8b).

**Additional file 16: Figure S9.** Phylogenetic tree constructed on the basis of 8 amino acid sequences belonging to Phenylalanine ammonia lyase (PAL) family proteins (Fig. S9a); Phylogenetic tree constructed on the basis of 8 amino acid sequences belonging to p-coumaroyl coenzyme A ligase (4-coumaroyl CoA ligase, 4CL) family proteins (Fig. S9b).

**Additional file 17: Table S8.** Gene-specific primers for qRT-PCR.

## Data Availability

The datasets supporting the conclusions of this article are included within the article and its additional files. The transcriptome raw data has been deposited in the Sequence Read Archive (SRA) database under the accession number PRJNA664220 (https://www.ncbi.nlm.nih.gov/bioproject/PRJNA664220).

## Abbreviations

LED: Light-emitting diodes
WL: White LED light
RL: Red LED light
BL: Blue LED light
DEGs: Differentially expressed genes
WG: Root tissue under WL
WJ: Stem tissue under WL
BY: Leaf tissue under BL
WY: Leaf tissue under WL
RY: Leaf tissue under RL
Vs.: Versus
GAP: Good Agricultural Practice
TFs: Transcription factors
MBW: MYB-bHLH-WD40
Nr: Non-redundant protein
Nt: NCBI nucleotide sequences
Pfam: The protein family
KOG: The euKaryotic Ortholog Groups
KEGG: The Kyoto Encyclopedia of Genes and Genomes
GO: The Gene Ontology
FPKM: Fragments per kilobase of transcript per million mapped transcript
PVC: Poly Vinyl Chloride
PPFD: Photosynthetic photon flux density
qRT-PCR: Quantitative Real-time PCR
PCA: Principal component analysis
HCA: Hierarchical cluster analysis
UV: Unit variance
PAL: Phenylalanine ammonia lyase
C4H: Cinnamate 4-hydroxylase
4CL: 4-coumaroyl CoA ligase
C3H: Coumarate 3-hydroxylase
HCT: Hydroxycinnamoyl-CoA shikimate hydroxycinnamoyl transferase
HQT: Hydroxyl-cinnamoyl CoA quinate hydroxycinnamoyl transferase
COMT: Caffeic acid 3-O-methyltransferase
CCoAOMT (CCOMT): Caffeoyl CoA 3-O-methyltransferase
F6’H: Feruloyl-CoA 6′-Hydroxylase
S8H: Scopoletin 8-hydroxylase
SGTF: Scopoletin glucosyltransferase
O-MT: O-methyltransferase
CHS: Chalcone synthase
CHI: Chalcone isomerase
F3H: Flavanone 3-hydroxylase
F3’H: Flavonoid 3′-hydroxylase
FLS: Flavonol synthase
UFGT: UDPG flavonoid O-glucosyltransferase
RT: Rhamnosyl transferase
DFR: Dihydroflavonol 4-reductase
LAR: Leucoanthocyanidin reductase
ANR: Anthocyanidin reductase
ANS or LDOX: Anthocyanin synthase or Leucoanthocyanidin dioxygenase
TAT: Tyrosine aminotransferase
HPPR: Hydroxyphenylpyruvate reductase
HPPD: 4-hydroxyphenylpyruvate dioxygenase
RAS: Rosmarinate synthase (rosmarinic acid synthase)
CYP (CPR): Cytochrome P450 reductase
CA2H: Caffeic acid 2-hydroxylase
PAs: Proanthocyanidins
r: Correlation coefficient
PAR: Photosynthetically active radiation
Phy: Phytochromes
Cry: Cryptochromes
Phot: Phototropins
COP1: CONSTITUTIVE PHOTOMORPHOGENCI 1
PIFs: PHYTOCHROME INTERACTING FACTORs
EBGs: Early biosynthetic genes
LBGs: Late biosynthetic genes
VIP: Variable importance projection

## References

[1] Li H, Gong X, Wang Z, Pan C, Zhao Y, Gao X, Liu W (2019). Multiple fingerprint profiles and chemometrics analysis of polysaccharides from *Sarcandra glabra*. Int J Biol Macromol.

[2] Zhou H, Liang J, Lv D, Hu Y, Zhu Y, Si J, Wu S (2013). Characterization of phenolics of *Sarcandra glabra* by non-targeted high-performance liquid chromatography fingerprinting and following targeted electrospray ionisation tandem mass spectrometry/time-of-flight mass spectrometry analyses. Food Chem.

[3] The state pharmacopoeia commission of People's Republic of China (2015). Pharmacopoeia of the People's Republic of China.

[4] Bourgaud F, Hehn A, Larbat R, Doerper S, Gontier E, Kellner S, Matern U (2006). Biosynthesis of coumarins in plants: a major pathway still to be unravelled for cytochrome P450 enzymes. Phytochem Rev.

[5] Liu J, Li X, Lin J, Li Y, Wang T, Jiang Q, Chen D (2016). *Sarcandra glabra* (Caoshanhu) protects mesenchymal stem cells from oxidative stress. A bioevaluation and mechanistic chemistry. BMC Complem Altern M.

[6] Cao H, Tan R, He R, Tang L, Wang X, Yao N, Duan W, Hu Y, Yao X, Kurihara H (2012). *Sarcandra glabra* extract reduces the susceptibility and severity of influenza in restraint-stressed mice. Evid Based Complement Alternat Med.

[7] Wei S, Chi J, Zhou M, Li R, Li Y, Luo J, Kong L (2019). Anti-inflammatory lindenane sesquiterpeniods and dimers from *Sarcandra glabra* and its upregulating AKT/Nrf2/HO-1 signaling mechanism. Ind Crop Prod.

[8] Guo X, Shen L, Tong Y, Zhang J, Wu G, He Q, Yu S, Ye X, Zou L, Zhang Z (2013). Antitumor activity of caffeic acid 3,4-dihydroxyphenethyl ester and its pharmacokinetic and metabolic properties. Phytomedicine..

[9] Li X, Zhang Y, Zeng X, Yang L, Deng Y (2011). Chemical profiling of bioactive constituents in *Sarcandra glabra* and its preparations using ultra-high-pressure liquid chromatography coupled with LTQ Orbitrap mass spectrometry. Rapid Commun Mass Spectrom.

[10] Zheng W, Wang S, Chen X, Hu Z (2003). Analysis of *Sarcandra glabra* and its medicinal preparations by capillary electrophoresis. Talanta..

[11] Yaermaimaiti S, Wang P, Luo J, Li R, Kong L (2016). Sesquiterpenoids from the seeds of *Sarcandra glabra* and the potential anti-inflammatory effects. Fitoterapia..

[12] Liao P, Liu P, Wang Y, Huang C, Lan L, Yang Y, Cui X (2018). Stereoscopic cultivation of *Panax notoginseng*: a new approach to overcome the continuous cropping obstacle. Ind Crop Prod.

[13] Ding J, Tu H, Zang Z, Huang M, Zhou S (2018). Precise control and prediction of the greenhouse growth environment of *Dendrobium candidum*. Comput Electron Agr.

[14] Yano A, Cossu M (2019). Energy sustainable greenhouse crop cultivation using photovoltaic technologies. Renew Sust Energ Rev.

[15] Tarakanov I, Yakovleva O, Konovalova I, Paliutina G, Anisimov A (2012). Light-emitting diodes: on the way to combinatorial lighting technologies for basic research and crop production. Acta Hortic.

[16] Yeh N, Chung J (2009). High-brightness LEDs—energy efficient lighting sources and their potential in indoor plant cultivation. Renew Sust Energ Rev.

[17] De Keyser E, Dhooghe E, Christiaens A, Van Labeke M, Van Huylenbroeck J (2019). LED light quality intensifies leaf pigmentation in ornamental pot plants. Sci Hortic.

[18] Berkovich YA, Konovalova IO, Smolyanina SO, Erokhin AN, Avercheva OV, Bassarskaya EM, Kochetova GV, Zhigalova TV, Yakovleva OS, Tarakanov IG (2017). LED crop illumination inside space greenhouses. Reach..

[19] Nanya K, Ishigami Y, Hikosaka S, Goto E (2012). Effects of blue and red light on stem elongation and flowering of tomato seedlings. Acta Hortic.

[20] Thwe AA, Kasemsap P, Vercambre G, Gay F, Phattaralerphong J, Gautier H (2020). Impact of red and blue nets on physiological and morphological traits, fruit yield and quality of tomato (*Solanum lycopersicum* Mill.). Sci Hortic.

[21] Hernández R, Kubota C (2016). Physiological responses of cucumber seedlings under different blue and red photon flux ratios using LEDs. Environ Exp Bot.

[22] Liu H, Chen Y, Hu T, Zhang S, Zhang Y, Zhao T, Yu H, Kang Y (2016). The influence of light-emitting diodes on the phenolic compounds and antioxidant activities in pea sprouts. J Funct Foods.

[23] Taulavuori K, Pyysalo A, Taulavuori E, Julkunen-Tiitto R (2018). Responses of phenolic acid and flavonoid synthesis to blue and blue-violet light depends on plant species. Environ Exp Bot.

[24] Liu Y, Fang S, Yang W, Shang X, Fu X (2018). Light quality affects flavonoid production and related gene expression in *Cyclocarya paliurus*. J Photoch Photobio B.

[25] Kapoor S, Raghuvanshi R, Bhardwaj P, Sood H, Saxena S, Chaurasia OP (2018). Influence of light quality on growth, secondary metabolites production and antioxidant activity in callus culture of *Rhodiola imbricata* Edgew. J Photoch Photobio B.

[26] Ni J, Dong L, Jiang Z, Yang X, Sun Z, Li J, Wu Y, Xu M (2018). Salicylic acid-induced flavonoid accumulation in *Ginkgo biloba* leaves is dependent on red and far-red light. Ind Crop Prod.

[27] Yang Q, Pan J, Shen G, Guo B (2019). Yellow light promotes the growth and accumulation of bioactive flavonoids in *Epimedium pseudowushanense*. J Photoch Photobio B.

[28] Fraser CM, Chapple C (2011). The Phenylpropanoid Pathway in *Arabidopsis*. Arabidopsis Book.

[29] Vogt T (2010). Phenylpropanoid biosynthesis. Mol Plant.

[30] Xu W, Grain D, Bobet S, Gourrierec J, Thévenin J, Kelemen Z, Lepiniec L, Dubos C (2014). Complexity and robustness of the flavonoid transcriptional regulatory network revealed by comprehensive analyses of MYB-bHLH-WDR complexes and their targets in *Arabidopsis* seed. New Phytol.

[31] Dubos C, Stracke R, Grotewold E, Weisshaar B, Martin C, Lepiniec L (2010). MYB transcription factors in *Arabidopsis*. Trends Plant Sci.

[32] Zimmermann IM, Heim MA, Weisshaar B, Uhrig JF (2004). Comprehensive identification of *Arabidopsis thaliana* MYB transcription factors interacting with R/B-like BHLH proteins. Plant J.

[33] Toledo-Ortiz G, Huq E, Quail PH (2003). The *Arabidopsis* basic/helix-loop-helix transcription factor family. Plant Cell.

[34] Smith TF, Gaitatzes C, Saxena K, Neer EJ (1999). The WD repeat: a common architecture for diverse functions. Trends Biochem Sci.

[35] Stracke R, Werber M, Weisshaar B (2001). The R2R3-MYB gene family in *Arabidopsis thaliana*. Curr Opin Plant Biol.

[36] Franklin KA, Quail PH (2010). Phytochrome functions in *Arabidopsis* development. J Exp Bot.

[37] Samuolienė G, Sirtautas R, Brazaitytė A, Sakalauskaitė J, Sakalauskienė S (2011). Duchovs.Kis P. the impact of red and blue light-emitting diode illumination on radish physiological indices. Cent Eur J of Biol.

[38] Huché-Thélier L, Crespel L, Gourrierec JL, Morel P, Sakr S, Leduc N (2016). Light signaling and plant responses to blue and UV radiations—perspectives for applications in horticulture. Environ Exp Bot.

[39] Hernández R, Kubota C (2015). Physiological responses of cucumber seedlings under different blue and red photon flux ratios using LEDs. Environ Exp Bot.

[40] Li J, Li G, Wang H, Wang DX (2011). Phytochrome signaling mechanisms. The Arabidopsis Book.

[41] Yu X, Liu H, Klejnot J, Lin C (2010). The Cryptochrome blue light receptors. The Arabidopsis Book.

[42] Christie JM (2007). Phototropin blue-light receptors. Annu Rev Plant Biol.

[43] Demotes-Mainard S, Péron T, Corot A, Bertheloot J, Le Gourrierec J, Pelleschi-Travier S, Crespel L, Morel P, Huché-Thélier L, Boumaza R (2016). Plant responses to red and far-red lights, applications in horticulture. Environ Exp Bot.

[44] Sellaro R, Hoecker U, Yanovsky M, Chory J, Casal JJ (2009). Synergism of red and blue light in the control of Arabidopsis gene expression and development. Curr Biol.

[45] Parks BM, Folta KM, Spalding EP (2001). Photocontrol of stem growth. Curr Opin Plant Biol.

[46] Koes R, Quattrocchio F, Mol J (1994). The flavonoid biosynthetic pathway in plants: function and evolution. Bioessays.

[47] Falcone Ferreyra ML, Rius SP, Casati P (2012). Flavonoids: biosynthesis, biological functions, and biotechnological applications. Front Plant Sci.

[48] Xu W, Dubos C, Lepiniec L (2015). Transcriptional control of flavonoid biosynthesis by MYB–bHLH–WDR complexes. Trends Plant Sci.

[49] Grotewold E (2005). Plant metabolic diversity: a regulatory perspective. Trends Plant Sci.

[50] Wang Z, Wang S, Xiao Y, Li Z, Wu M, Xie X, Li H, Mu W, Li F, Liu P (2020). Functional characterization of a HD-ZIP IV transcription factor NtHDG2 in regulating flavonols biosynthesis in *Nicotiana tabacum*. Plant Physiol Bioch.

[51] Owens D, Alerding A, Crosby K, Bandara A, Westwood J, Winkel B (2008). Functional analysis of a predicted Flavonol synthase gene family in *Arabidopsis*. Plant Physiol.

[52] Lukacin R, Wellmann F, Britsch L, Martens S, Matern U (2003). Flavonol synthase from *Citrus unshiu* is a bifunctional dioxygenase. Phytochemistry.

[53] Huang J, Gu M, Lai Z, Fan B, Shi K, Zhou Y, Yu J, Chen Z (2010). Functional analysis of the *Arabidopsis* PAL gene family in plant growth, development, and response to environmental stress. Plant Physiol.

[54] Ehlting J, Büttner D, Wang Q, Douglas CJ, Kombrink E (1999). Three 4-coumarate: coenzyme a ligases in *Arabidopsis thaliana* represent two evolutionarily divergent classes in angiosperms. Plant J.

[55] Siwinska J, Siatkowska K, Olry A, Grosjean J, Ihnatowicz A (2018). Scopoletin 8-hydroxylase: a novel enzyme involved in coumarin biosynthesis and iron-deficiency responses in *Arabidopsis*. J Exp Bot.

[56] Carretero-Paulet L, Galstyan A, Roig-Villanova I, Martínez-García JF, Bilbao-Castro JR, Robertson DL (2010). Genome-wide classification and evolutionary analysis of the bHLH family of transcription factors in *Arabidopsis*, poplar, Rice, Moss, and algae. Plant Physiol.

[57] Ambawat S, Sharma P, Yadav NR, Yadav RC (2013). MYB transcription factor genes as regulators for plant responses: an overview. Physiol Mol Biol Pla.

[58] Liu J, Osbourn A, Ma P (2015). MYB transcription factors as regulators of phenylpropanoid metabolism in plants. Mol Plant.

[59] Bipei Z, Martin H (2019). Evolutionary Analysis of MBW Function by phenotypic rescue in *Arabidopsis thaliana*. Front Plant Sci.

[60] Feller A, Machemer K, Braun EL, Grotewold E (2011). Evolutionary and comparative analysis of MYB and bHLH plant transcription factors. Plant J.

[61] Ramsay N, Glover B (2005). MYB-bHLH-WD40 protein complex and the evolution of cellular diversity. Trends Plant Sci.

[62] Stracke R, Jahns O, Keck M, Tohge T, Niehaus K, Fernie AR, Weisshaar B (2010). Analysis of production of flavonol glycosides-dependent flavonol glycoside accumulation in *Arabidopsis thaliana* plants reveals MYB11-, MYB12- and MYB111-independent flavonol glycoside accumulation. New Phytol.

[63] Gonzalez A, Zhao M, Leavitt JM, Lloyd AM (2008). Regulation of the anthocyanin biosynthetic pathway by the TTG1/bHLH/Myb transcriptional complex in *Arabidopsis* seedlings. Plant J.

[64] Grabherr M, Haas B, Yassour M, Levin J, Thompson D, Amit I, Adiconis X, Fan L, Raychowdhury R, Zeng Q (2011). Full-length transcriptome assembly from RNA-Seq data without a reference genome. Nat Biotechnol.

[65] Love MI, Huber W, Anders S (2014). Moderated estimation of fold change and dispersion for RNA-seq data with DESeq2. Genome Biol.

[66] Young MD, Wakefield MJ, Smyth GK, Oshlack A (2010). Gene ontology analysis for RNA-seq: accounting for selection bias. Genome Biol.

[67] Chen C, Chen H, Zhang Y, Thomas HR, Frank MH, He Y, Xia R (2020). TBtools: an integrative toolkit developed for interactive analyses of big biological data. Mol Plant.

[68] Pérez-Rodríguez P, Riaño-Pachón DM, Corrêa LGG, Rensing SA, Kersten B, Mueller-Roeber B (2010). PlnTFDB: updated content and new features of the plant transcription factor database. Nucleic Acids Res.

[69] Atchley WR, Terhalle W, Dress A (1999). Positional dependence, cliques, and predictive motifs in the bHLH protein domain. J Mol Evol.

[70] Sudhir K, Glen S, Li M, Christina K, Koichiro T (2018). MEGA X: Molecular evolutionary genetics analysis across computing platforms. Mol Biol Evol.

