# Supplementary material for: Transcriptomic and metabolomic profiling reveals the effect of LED light quality on morphological traits, and phenylpropanoid-derived compounds accumulation in Sarcandra glabra seedlings
Source: BMC Plant Biol. 2020 Oct 15;20:476. doi: 10.1186/s12870-020-02685-w (PMC7574309; doi:10.1186/s12870-020-02685-w)
Supplement: Supplementary file 2 — Additional file 2: Table S2. The statistics of the de novo assembly based on RNA-seq data. [file 12870_2020_2685_MOESM2_ESM.doc]

**Table S2 The statistics of the *de novo* assembly based on RNA-seq data**

| Category | Transcript | Unigene |
| --- | --- | --- |
| Total number ( ≥ 300 bp) | 281,381 | 129,934 |
| ≥ 500 bp | 190,835 | 81,140 |
| ≥ 2000 bp | 52,678 | 17,374 |
| Mean length (bp) | 1255 | 1061 |
| Max length (bp) | 19,022 | 19,022 |
| N50 | 2014 | 1622 |
| N90 | 503 | 442 |
