# Supplementary material for: Transcriptomic and metabolomic profiling reveals the effect of LED light quality on morphological traits, and phenylpropanoid-derived compounds accumulation in Sarcandra glabra seedlings
Source: BMC Plant Biol. 2020 Oct 15;20:476. doi: 10.1186/s12870-020-02685-w (PMC7574309; doi:10.1186/s12870-020-02685-w)
Supplement: Supplementary file 3 — Additional file 3: Table S3. The statistics of the annotated unigenes in seven databases. [file 12870_2020_2685_MOESM3_ESM.doc]

| Database | Number of unigenes | Percentage (%) |
| --- | --- | --- |
| Annotated in Nr | 47736 | 36.73 |
| Annotated in Nt | 22349 | 17.20 |
| Annotated in KO | 14546 | 11.19 |
| Annotated in Swiss-Prot | 28878 | 22.22 |
| Annotated in Pfam | 33626 | 25.87 |
| Annotated in GO | 33626 | 25.87 |
| Annotated in KOG | 6915 | 5.32 |
| Annotated in all databases | 3932 | 3.02 |
| Annotated in at least one database | 60970 | 46.92 |
| Total unigene | 129934 | 100.00 |

**Table S3 The statistics of the annotated unigenes in seven databases**
