# Supplementary figures and images for: Transcriptomic and metabolomic profiling reveals the effect of LED light quality on morphological traits, and phenylpropanoid-derived compounds accumulation in Sarcandra glabra seedlings
Source: BMC Plant Biol. 2020 Oct 15;20:476. doi: 10.1186/s12870-020-02685-w (PMC7574309; doi:10.1186/s12870-020-02685-w)

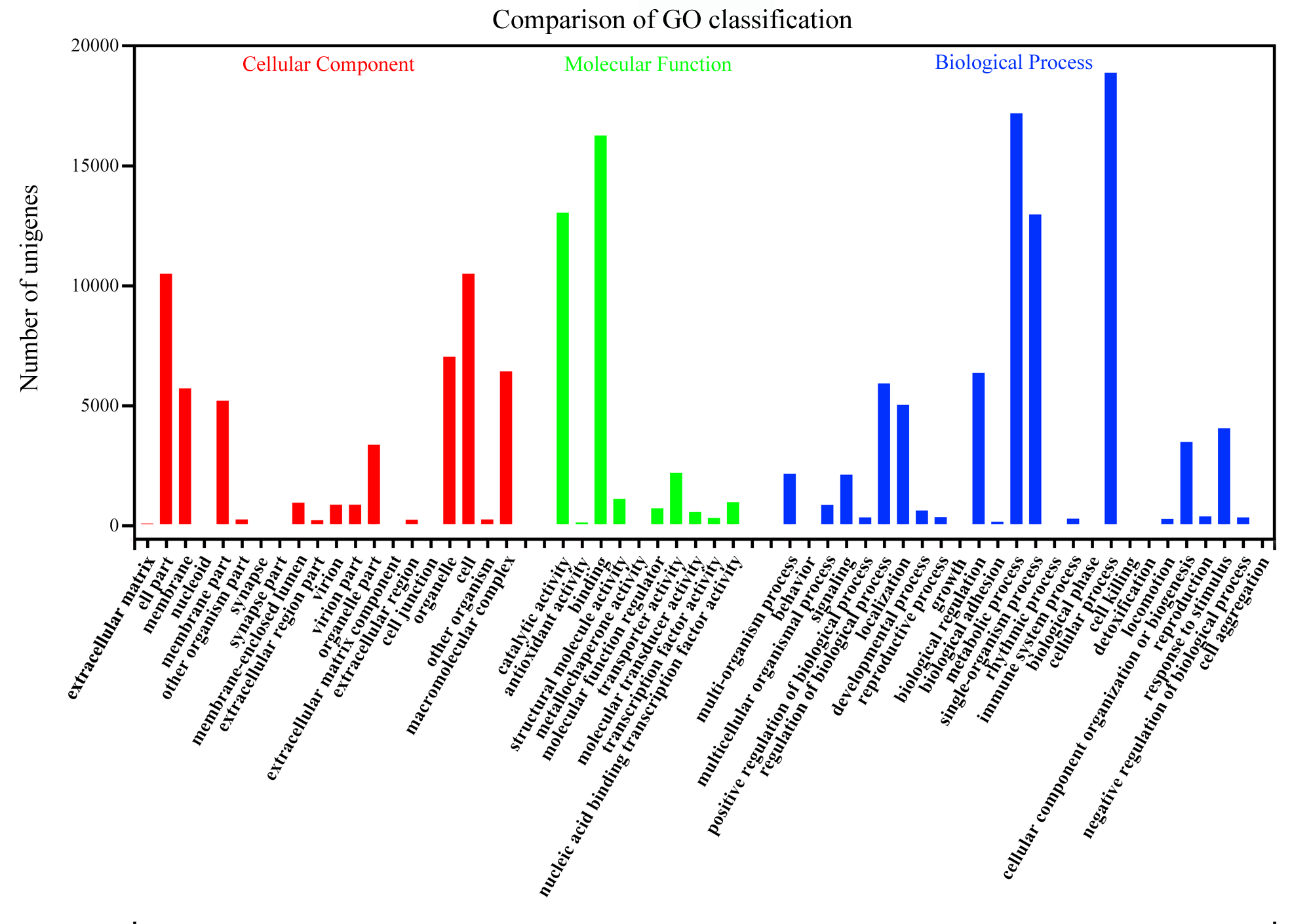


**Fig. S1 The histogram of GO annotation of all unigenes**

Supplement: Supplementary file 4 — Additional file 4: Figure S1. The histogram of GO annotation of all unigenes. [file 12870_2020_2685_MOESM4_ESM.doc]
