# Supplementary material for: Transcriptomic and metabolomic profiling reveals the effect of LED light quality on morphological traits, and phenylpropanoid-derived compounds accumulation in Sarcandra glabra seedlings
Source: BMC Plant Biol. 2020 Oct 15;20:476. doi: 10.1186/s12870-020-02685-w (PMC7574309; doi:10.1186/s12870-020-02685-w)
Supplement: Supplementary file 5 — Additional file 5: Figure S2. Enriched GO Terms among BY vs. RY (Fig.S2a), BY vs. WY (Fig.S2b), WG vs. WY (Fig.S2c), WJ vs. WG (Fig.S2d), and WJ vs. WY. (Fig. S2e) groups. [file 12870_2020_2685_MOESM5_ESM.doc]

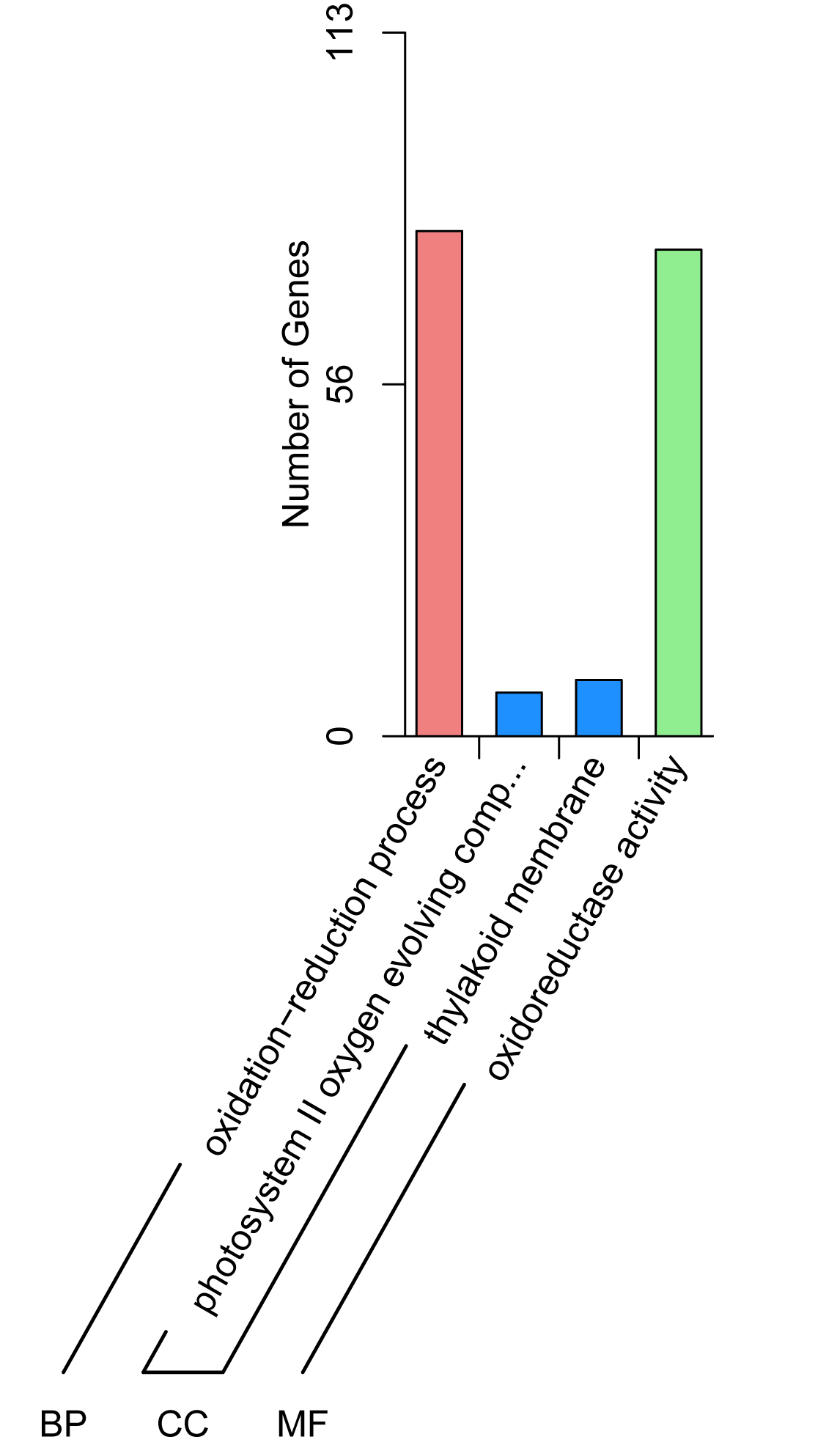


**Fig. S2a Enriched GO Terms among BY vs. RY**


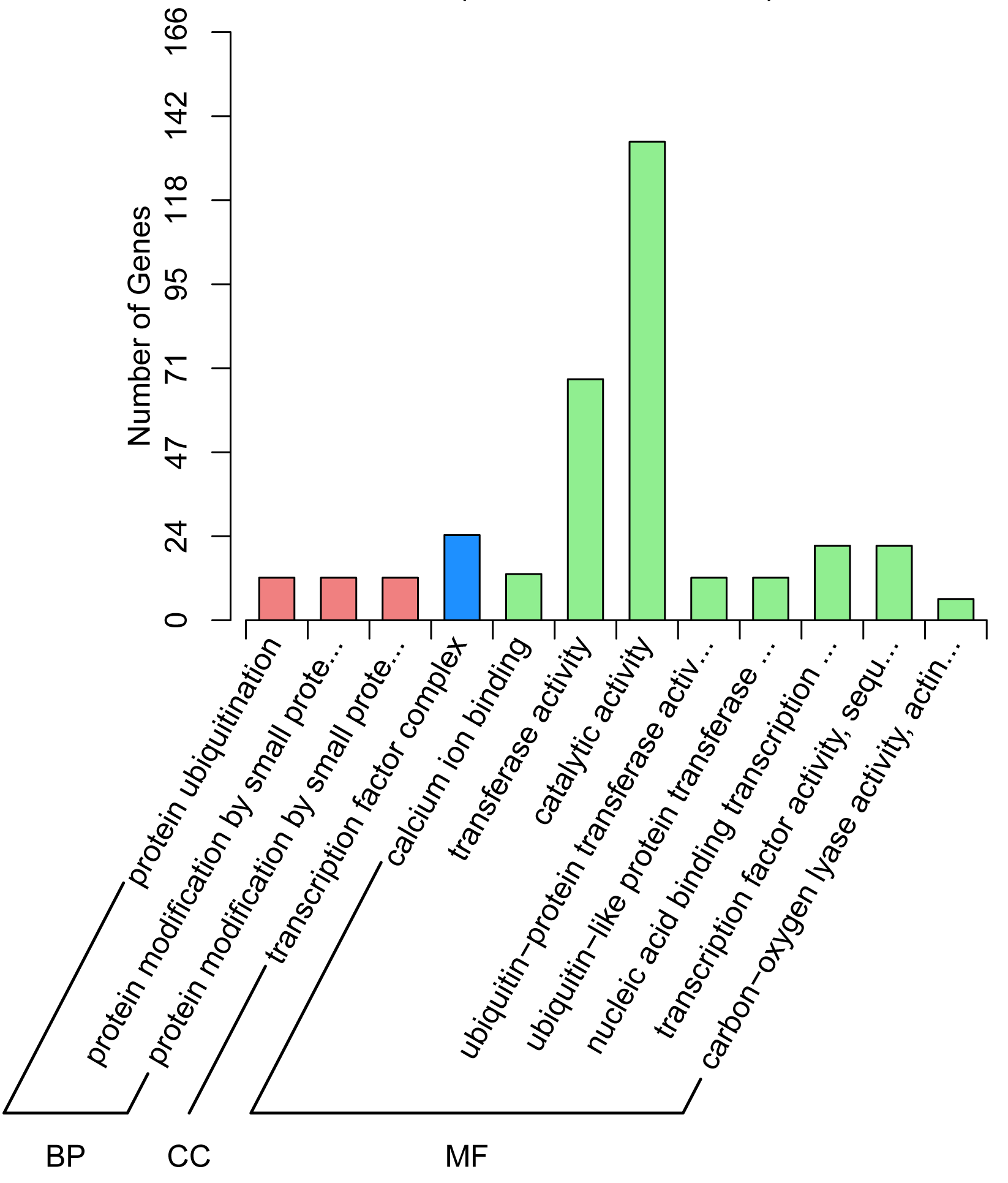


**Fig. S2b Enriched GO Terms among BY vs. WY**


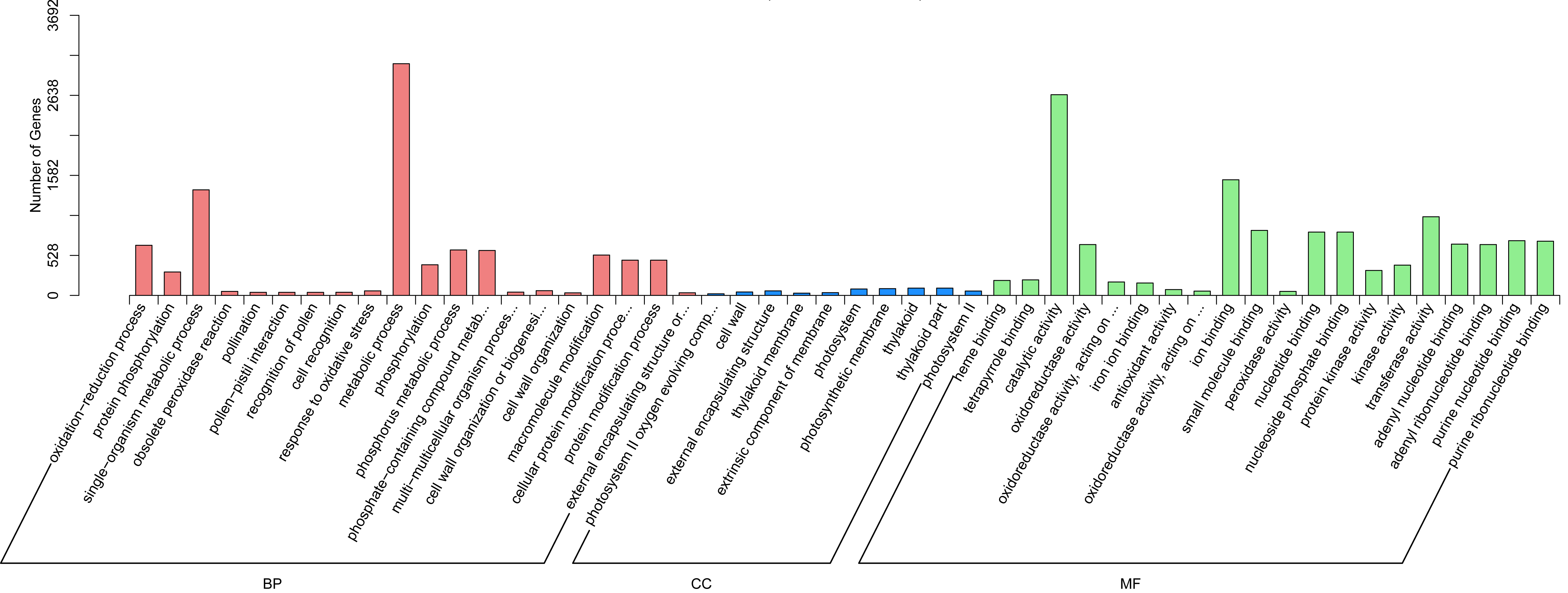


**Fig. S2c Enriched GO Terms among WG vs. WY**


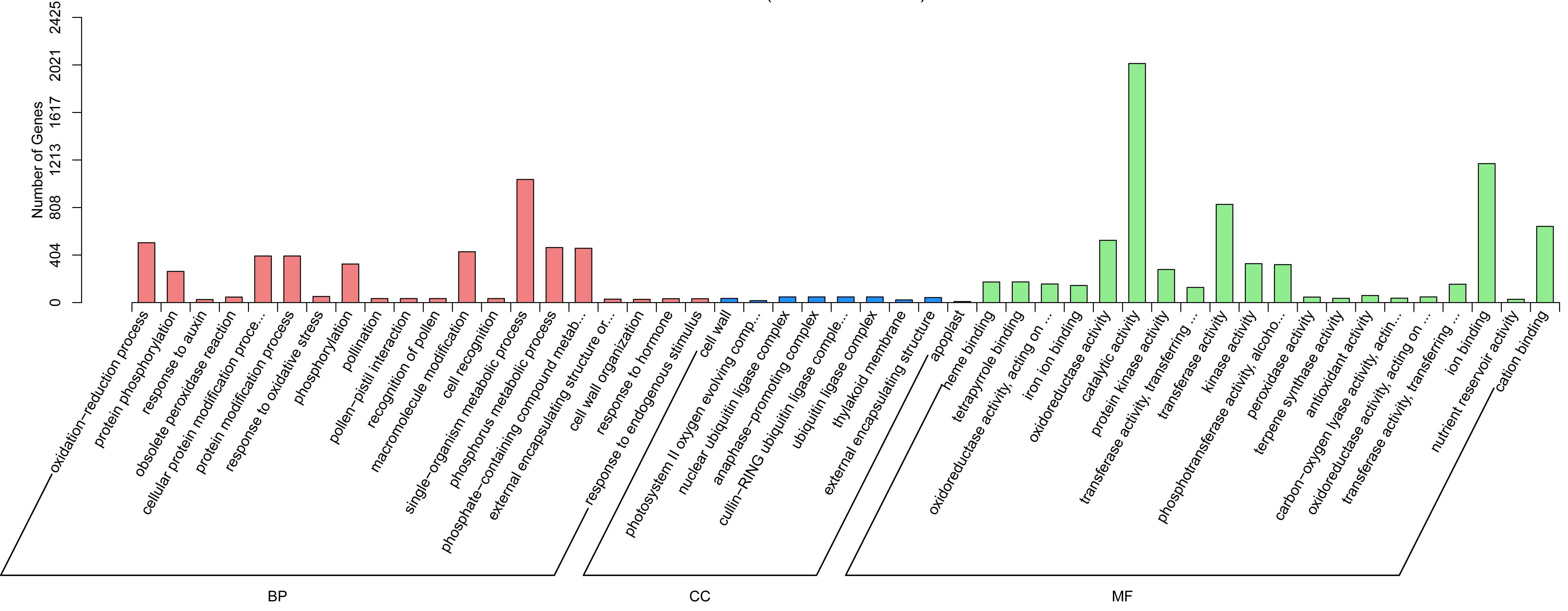


**Fig. S2d Enriched GO Terms among WJ vs. WG**


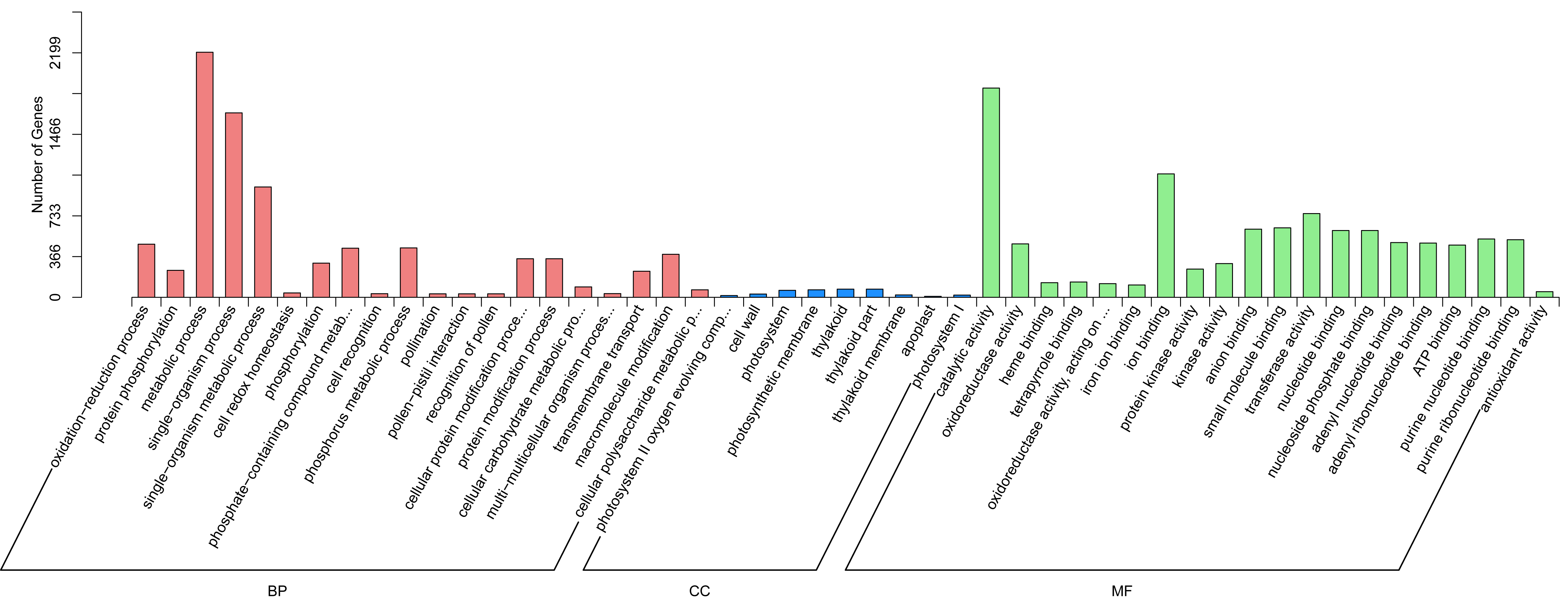


**Fig. S2e Enriched GO Terms among WJ vs. WY**
