# Supplementary material for: Transcriptomic and metabolomic profiling reveals the effect of LED light quality on morphological traits, and phenylpropanoid-derived compounds accumulation in Sarcandra glabra seedlings
Source: BMC Plant Biol. 2020 Oct 15;20:476. doi: 10.1186/s12870-020-02685-w (PMC7574309; doi:10.1186/s12870-020-02685-w)
Supplement: Supplementary file 6 — Additional file 6: Figure S3. KEGG enrichment among BY vs. RY (Fig.S3a), BY vs. WY (Fig.S3b), RY vs. WY (Fig.S3c), WG vs. WY (Fig.S3d), WJ vs. WG (Fig.S3e), and WJ vs. WY (Fig.S3f) groups. [file 12870_2020_2685_MOESM6_ESM.doc]

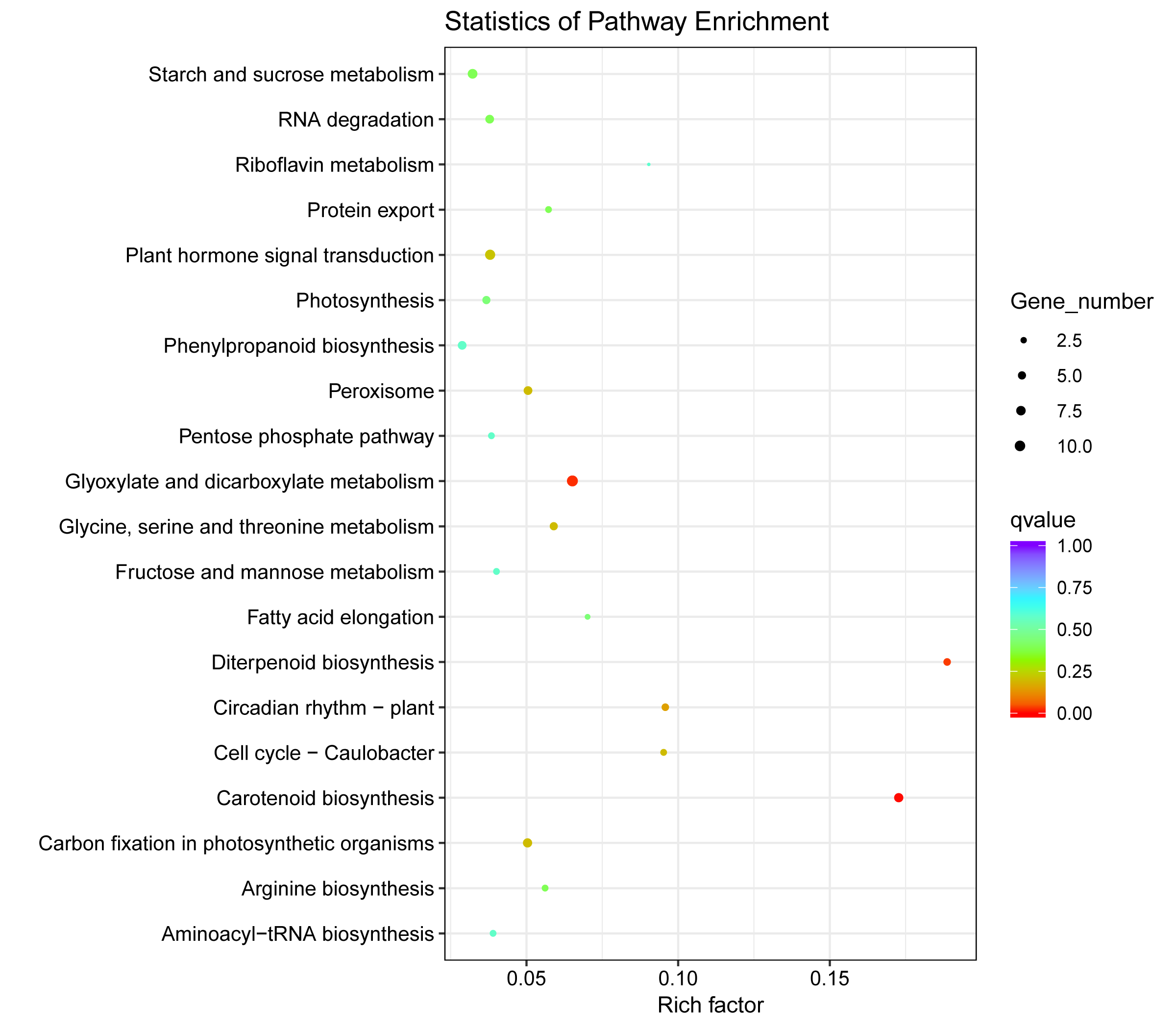


**Fig. S3a KEGG enrichment among BY vs. RY**


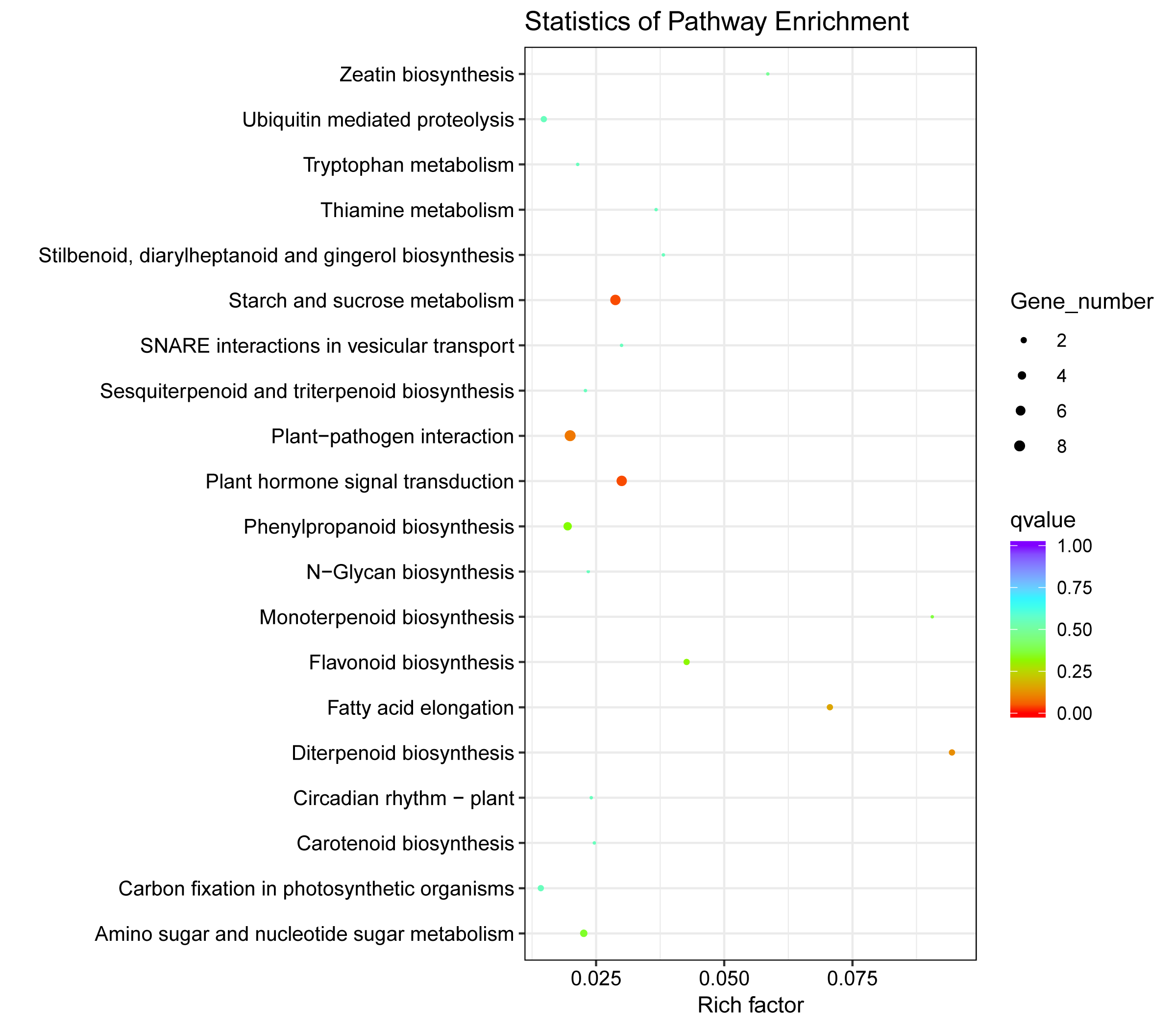


**Fig. S3b KEGG enrichment among BY vs. WY**


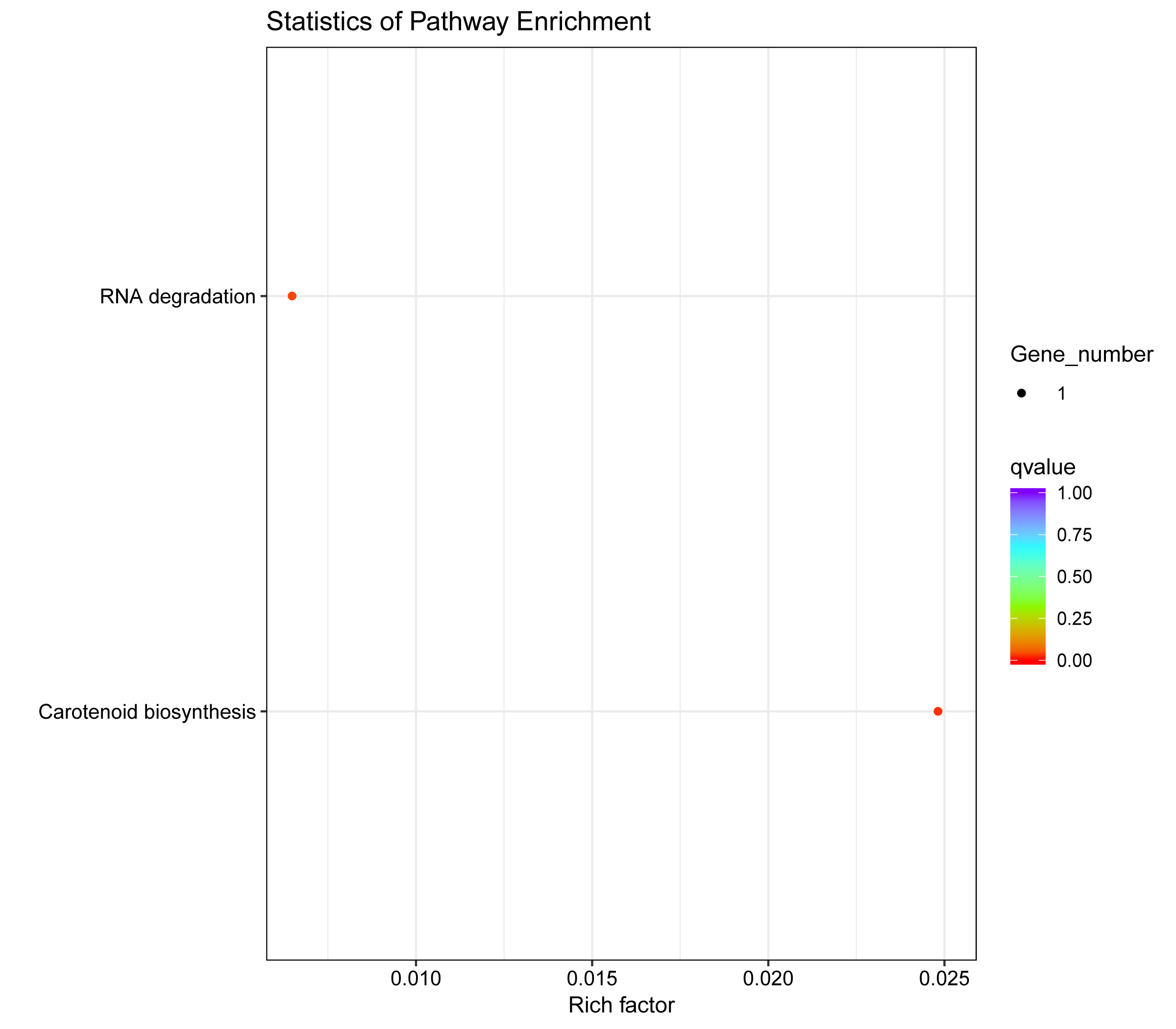


**Fig. S3c KEGG enrichment among RY vs. WY**


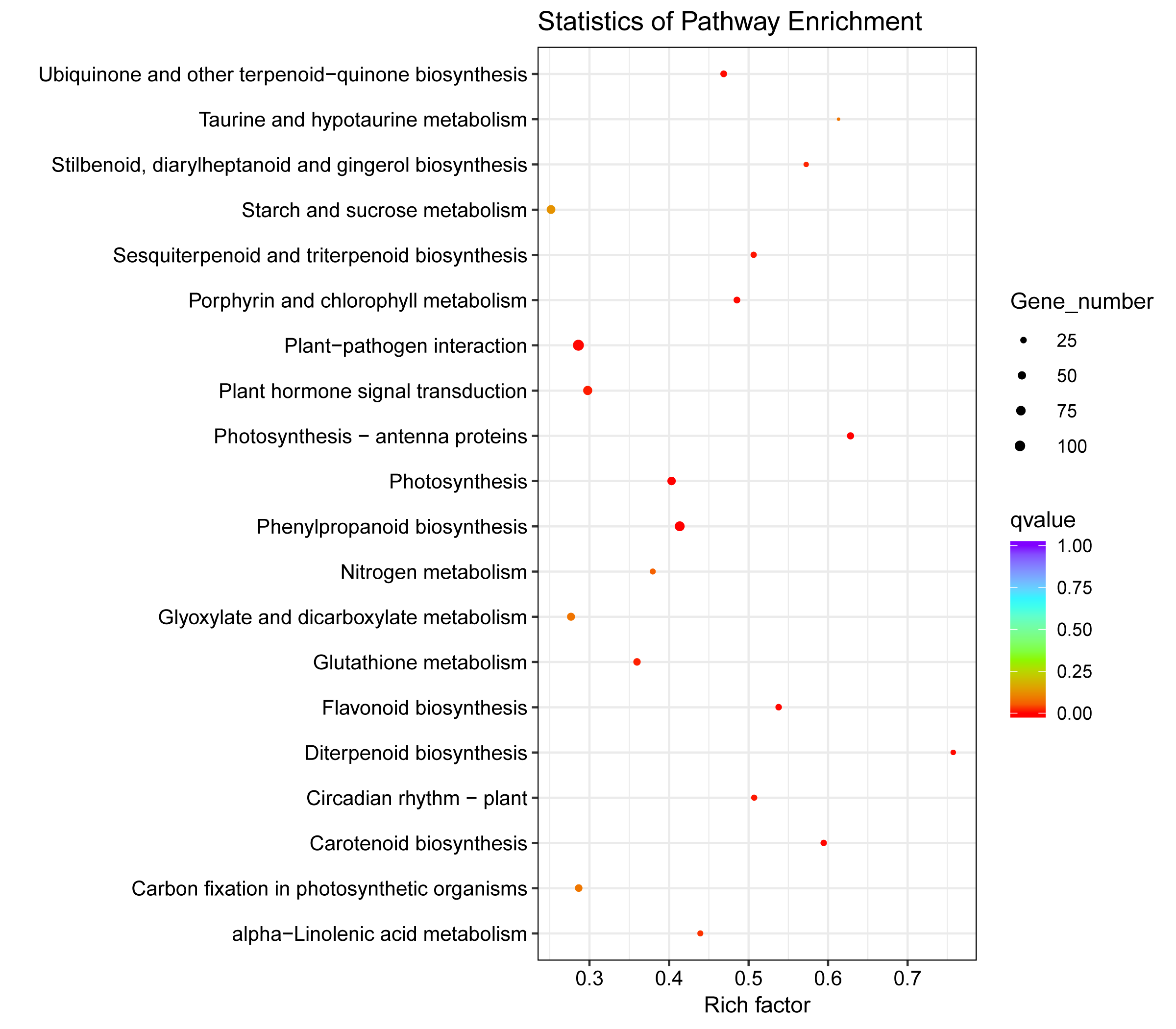


**Fig. S3d KEGG enrichment among WG vs.WY**


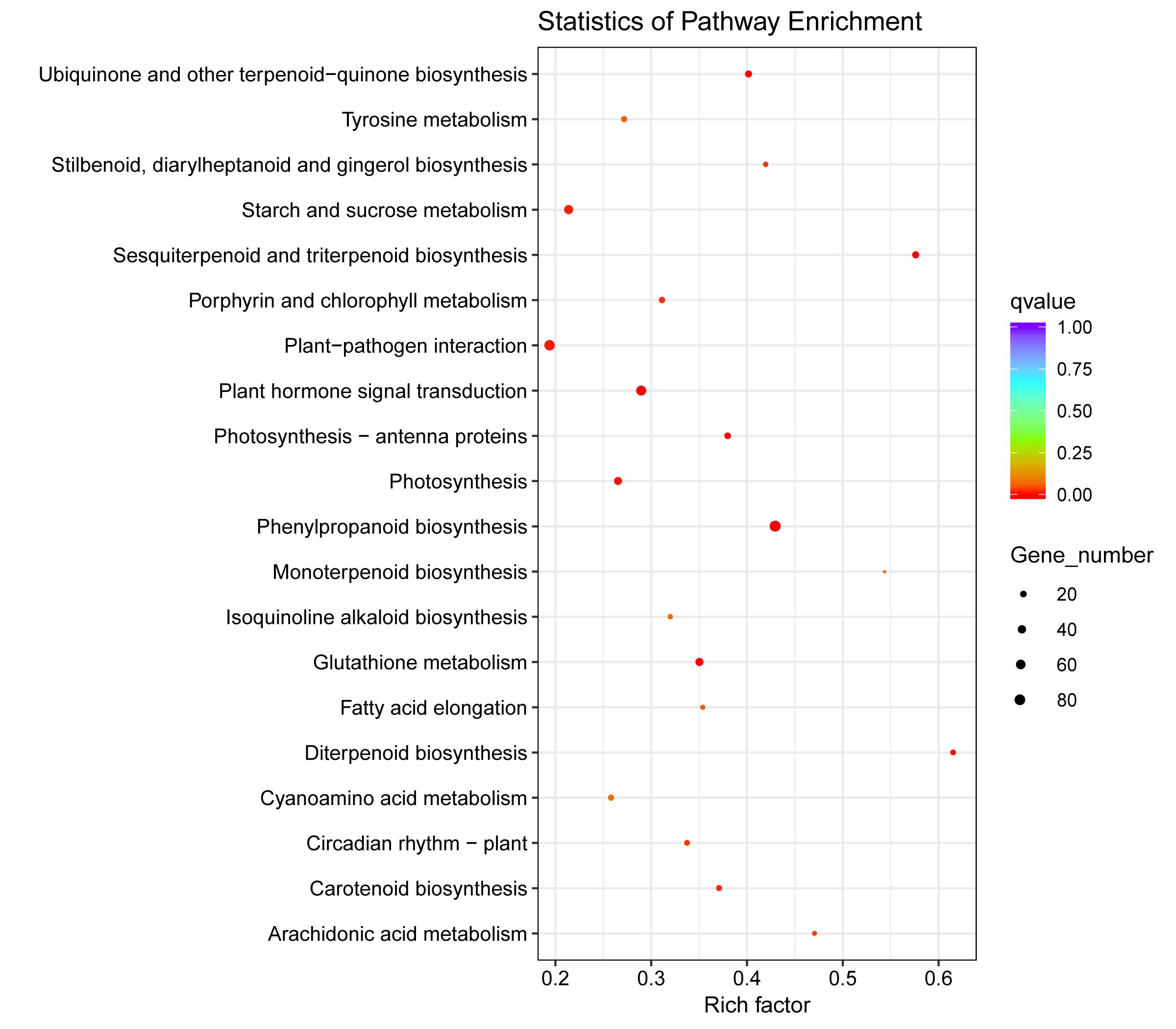


**Fig. S3e KEGG enrichment among WJ vs. WG**


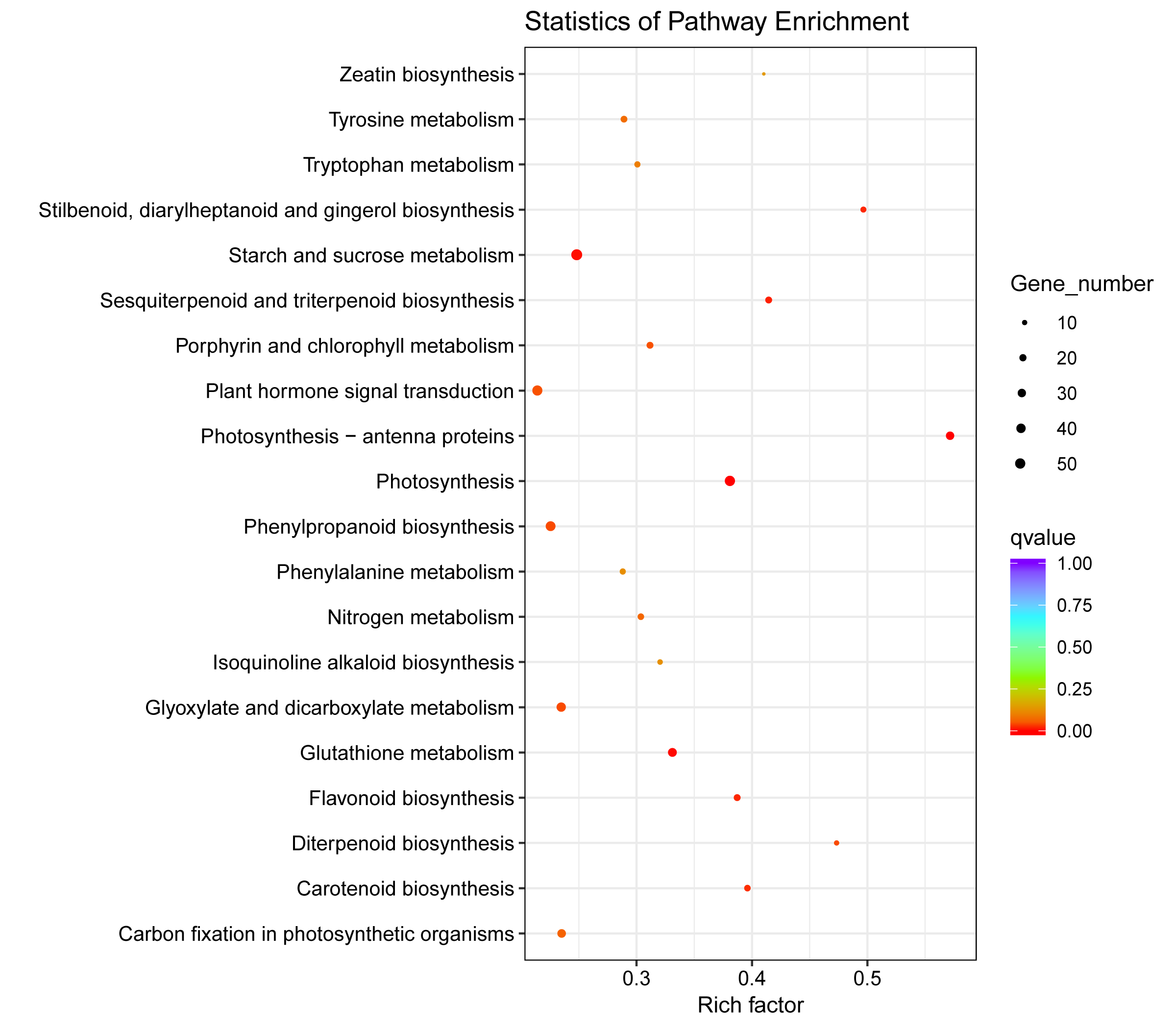


**Fig. S3f KEGG enrichment among WJ vs. WY**
