# Supplementary material for: Transcriptomic and metabolomic profiling reveals the effect of LED light quality on morphological traits, and phenylpropanoid-derived compounds accumulation in Sarcandra glabra seedlings
Source: BMC Plant Biol. 2020 Oct 15;20:476. doi: 10.1186/s12870-020-02685-w (PMC7574309; doi:10.1186/s12870-020-02685-w)
Supplement: Supplementary file 10 — Additional file 10: Figure S4. Phylogenetic tree constructed on the basis of 19 amino acid sequences belonging to 2-oxoglutarate (2OG) and Fe (II)-dependent oxygenase superfamily proteins. [file 12870_2020_2685_MOESM10_ESM.doc]

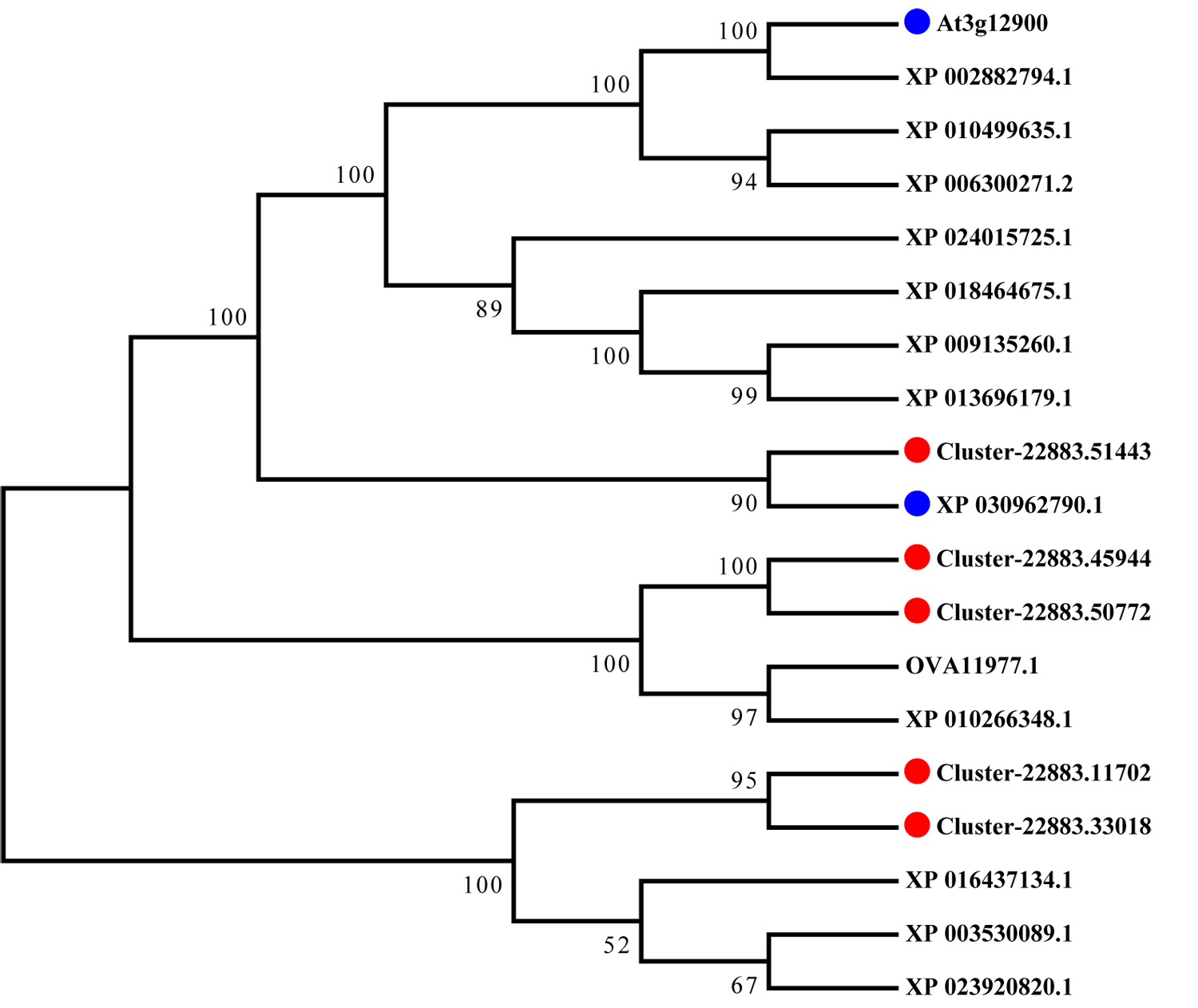


**Fig. S4 Phylogenetic tree constructed on the basis of 19 amino acid sequences belonging to 2-oxoglutarate (2OG) and Fe (II)-dependent oxygenase superfamily proteins.** Bootstrap values are displayed as percentages (1000 replicates) when greater than 50% at the branches. The red circles showed the putative Feruloyl-CoA 6´-Hydroxylases or scopoletin 8-hydroxylase from *S. glabra.* The blue circles represented the scopoletin 8-hydroxylase from other plant organisms. At3g12900: scopoletin 8-hydroxylase from *Arabidopsis thaliana*. XP_030962790.1: scopoletin 8-hydroxylase-like from *Quercus lobata*. XP_002882794.1: feruloyl CoA ortho-hydroxylase 2 from *Arabidopsis lyrata* subsp. lyrata. XP_010499635.1: feruloyl CoA ortho-hydroxylase 2 from *Camelina sativa*. XP_006300271.2: feruloyl CoA ortho-hydroxylase 2 from *Capsella rubella*. XP_024015725.1: feruloyl CoA ortho-hydroxylase 2 from *Eutrema salsugineum*. XP_009135260.1: feruloyl CoA ortho-hydroxylase 2-like from *Brassica rapa*. XP_013696179.1: feruloyl CoA ortho-hydroxylase 2 from *Brassica napus*. XP_018464675.1: feruloyl CoA ortho-hydroxylase 2-like from *Raphanus sativus*. XP_003530089.1: feruloyl CoA ortho-hydroxylase 1 from Glycine max. XP_023920820.1: feruloyl CoA ortho-hydroxylase 1-like from *Quercus suber*. XP_016437134.1: feruloyl CoA ortho-hydroxylase 2-like from *Nicotiana tabacum*. OVA11977.1: Oxoglutarate/iron-dependent dioxygenase from *Macleaya cordata*. XP_010266348.1: feruloyl CoA ortho-hydroxylase 2-like from *Nelumbo nucifera*.
