# Supplementary material for: Transcriptomic and metabolomic profiling reveals the effect of LED light quality on morphological traits, and phenylpropanoid-derived compounds accumulation in Sarcandra glabra seedlings
Source: BMC Plant Biol. 2020 Oct 15;20:476. doi: 10.1186/s12870-020-02685-w (PMC7574309; doi:10.1186/s12870-020-02685-w)
Supplement: Supplementary file 11 — Additional file 11: Figure S5. Phylogenetic tree constructed on the basis of 24 amino acid sequences belonging to hydroxycinnamoyltransferases. [file 12870_2020_2685_MOESM11_ESM.doc]

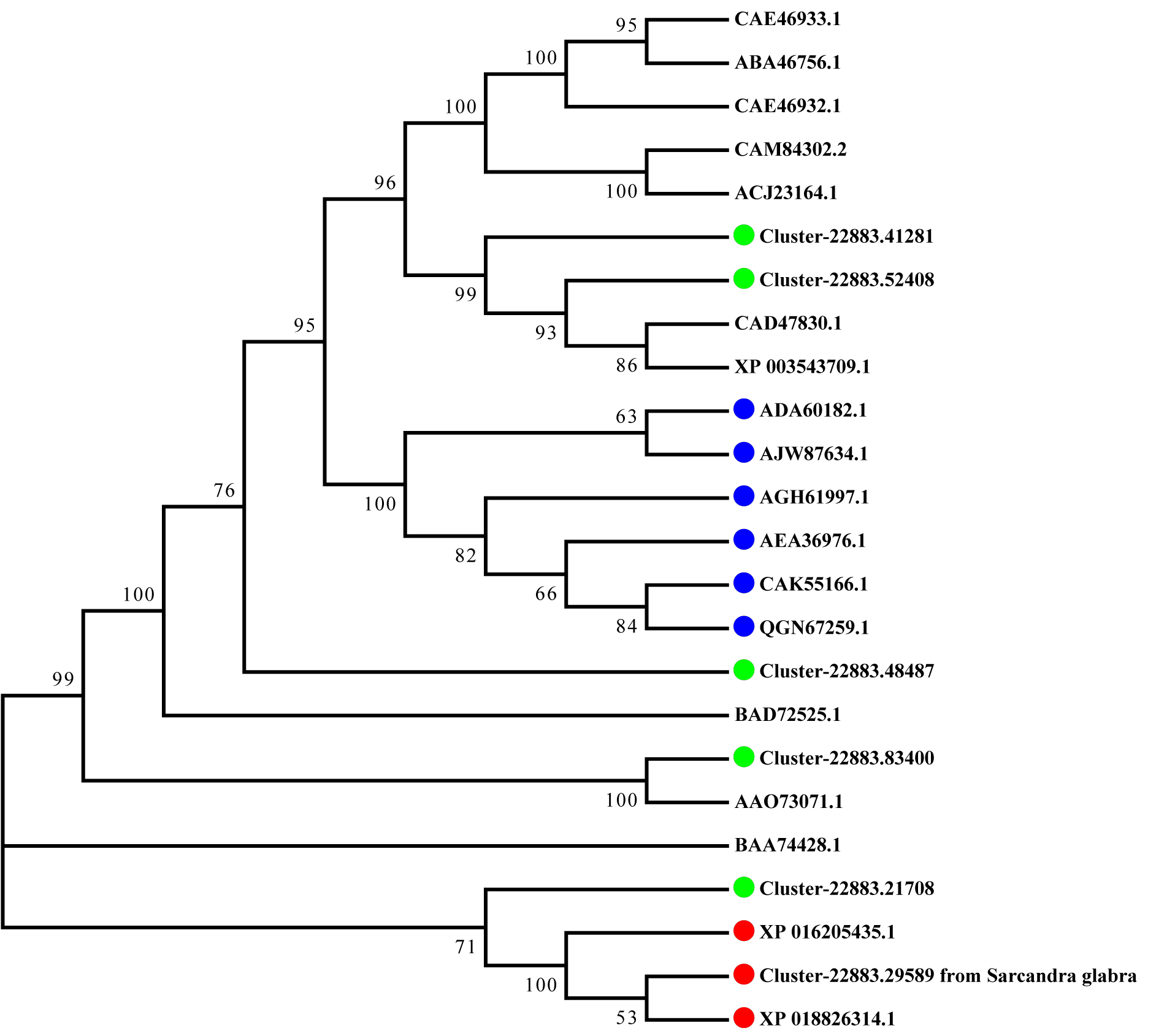


**Fig. S5 Phylogenetic tree constructed on the basis of 24 amino acid sequences belonging to hydroxycinnamoyltransferases.** Bootstrap values are displayed as percentages (1000 replicates) when greater than 50% at the branches. The 5 green circles represented the cloned full-length amino acids sequences encoding hydroxycinnamoyltransferases from *S. glabra*. The 3 red circles displayed the predicted rosmarinate synthase. Among of them, the middle sequences (Cluster-22883.29589) might encode the rosmarinate synthase from *S. glabra*. XP_018826314.1: predicted rosmarinate synthase-like from *Juglans regia.* XP_016205435.1: rosmarinate synthase from *Arachis ipaensis.* The 6 blue circles indicated the rosmarinic acid synthase. CAK55166.1: rosmarinic acid synthase from *Coleus blumei*. QGN67259.1: rosmarinic acid synthase from *Ocimum tenuiflorum*. AEA36976.1: rosmarinic acid synthase from *Lavandula angustifolia*. ADA60182.1: rosmarinic acid synthase from *Salvia miltiorrhiza*. AGH61997.1: rosmarinic acid synthase from *Perilla frutescens*. AJW87634.1: rosmarinic acid synthase from *Prunella vulgaris*. CAM84302.2: hydroxycinnamoyl CoA quinate transferase from *Cynara cardunculus* var. scolymus. ACJ23164.1: hydroxycinnamoyl CoA quinate transferase 2 from *Cynara cardunculus* var. scolymus. CAE46933.1: hydroxycinnamoyl CoA quinate transferase from *Solanum lycopersicum*. ABA46756.1: hydroxycinnamoyl-CoA quinate-like protein from *Solanum tuberosum*. CAD47830.1: hydroxycinnamoyl transferase from *Nicotiana tabacum*. CAE46932.1: hydroxycinnamoyl CoA quinate transferase from *Nicotiana tabacum*. BAD72525.1: putative hydroxycinnamoyl CoA quinate transferase from *Oryza sativa*. AAO73071.1: agmatine coumaroyltransferase from *Hordeum vulgare*. BAA74428.1: Anthocyanin 5-aromatic acyltransferase from *Gentiana triflora*. XP_003543709.1: shikimate O-hydroxycinnamoyltransferase from *Glycine max*.
