# Supplementary material for: Transcriptomic and metabolomic profiling reveals the effect of LED light quality on morphological traits, and phenylpropanoid-derived compounds accumulation in Sarcandra glabra seedlings
Source: BMC Plant Biol. 2020 Oct 15;20:476. doi: 10.1186/s12870-020-02685-w (PMC7574309; doi:10.1186/s12870-020-02685-w)
Supplement: Supplementary file 12 — Additional file 12: Figure S6. The primary structure of 53 R2R3-MYB domains in S. glabra. [file 12870_2020_2685_MOESM12_ESM.doc]

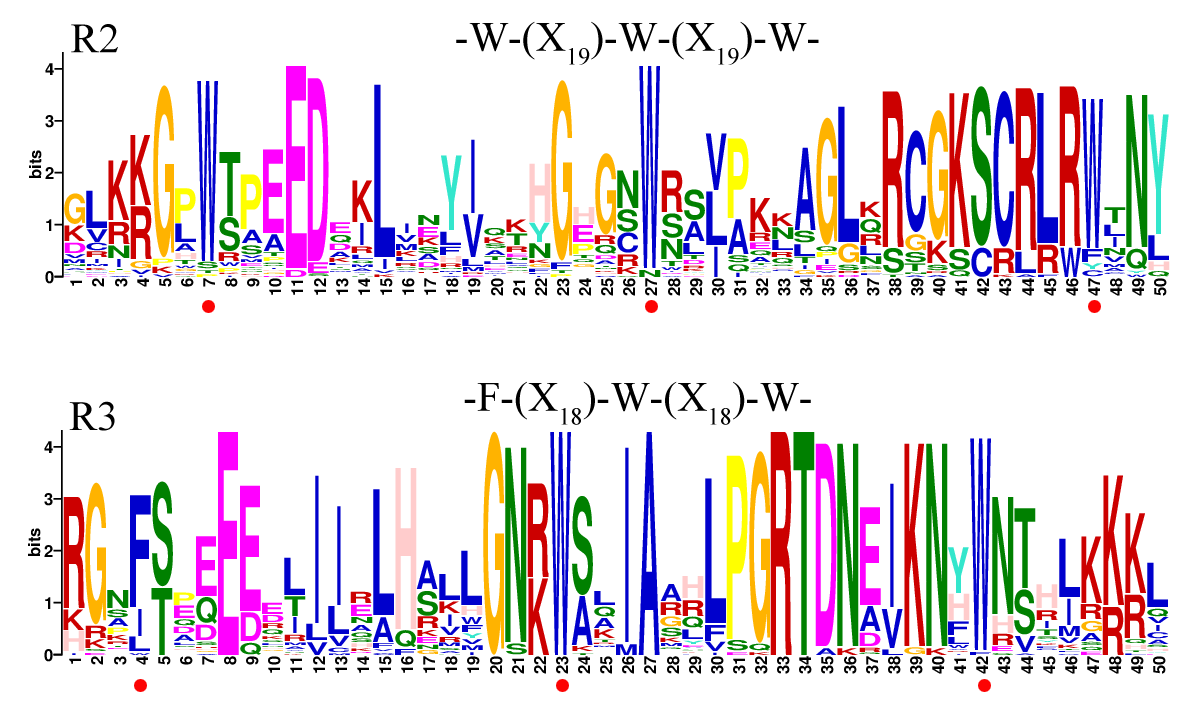


**Fig. S6 The primary structure of 53 R2R3-MYB domains in *S. glabra.*** R2, R2-type MYB repeat; R3, R3-type MYB repeat;W, tryptophan; X, amino acid; F, phenylalanine.conservative.
