# Supplementary material for: Transcriptomic and metabolomic profiling reveals the effect of LED light quality on morphological traits, and phenylpropanoid-derived compounds accumulation in Sarcandra glabra seedlings
Source: BMC Plant Biol. 2020 Oct 15;20:476. doi: 10.1186/s12870-020-02685-w (PMC7574309; doi:10.1186/s12870-020-02685-w)
Supplement: Supplementary file 13 — Additional file 13: Figure S7. Multiple sequence alignment of bHLH domains of the 53 members of the SgbHLH proteins family. [file 12870_2020_2685_MOESM13_ESM.doc]

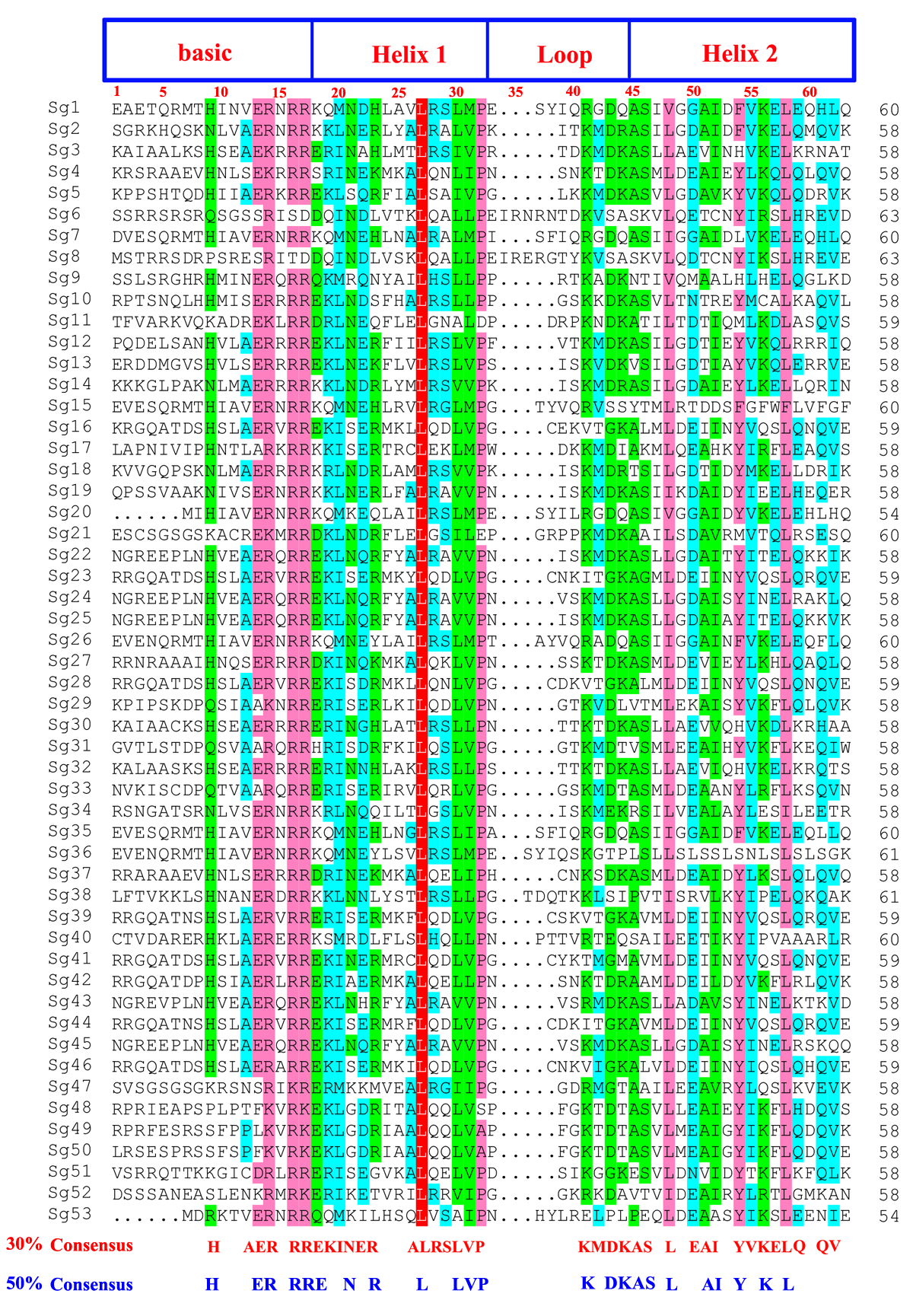


**Fig. S7 Multiple sequence alignment of bHLH domains of the 53 members of the SgbHLH proteins family.**

On the left column, we have assigned each SgbHLH protein a number in sequence (1 to 53) in this study. The scheme at top showed the location and boundaries of the basic, helix, and loop regions within the SgbHLH domain. On the right column, the number of every row indicates the total amino acids of the defined SgbHLH domains. The shading of the alignment presents identical residues in red, conserved residues in pink and blue, and similar residues in green. circles denote gaps. The SgbHLH consensus motif at bottom is based on the residues with 30 % (in red) and 50 % (in blue)conservation among the 53 proteins shown.
