# Supplementary material for: Transcriptomic and metabolomic profiling reveals the effect of LED light quality on morphological traits, and phenylpropanoid-derived compounds accumulation in Sarcandra glabra seedlings
Source: BMC Plant Biol. 2020 Oct 15;20:476. doi: 10.1186/s12870-020-02685-w (PMC7574309; doi:10.1186/s12870-020-02685-w)
Supplement: Supplementary file 15 — Additional file 15: Figure S8. Phylogenetic tree constructed on the basis of 11 amino acid sequences belonging to Cryptochromes (Cry) family proteins (Fig.S8a); Phylogenetic tree constructed on the basis of 6 amino acid sequences belonging to Phototropin (Phot) family proteins (Fig. S8b). [file 12870_2020_2685_MOESM15_ESM.doc]

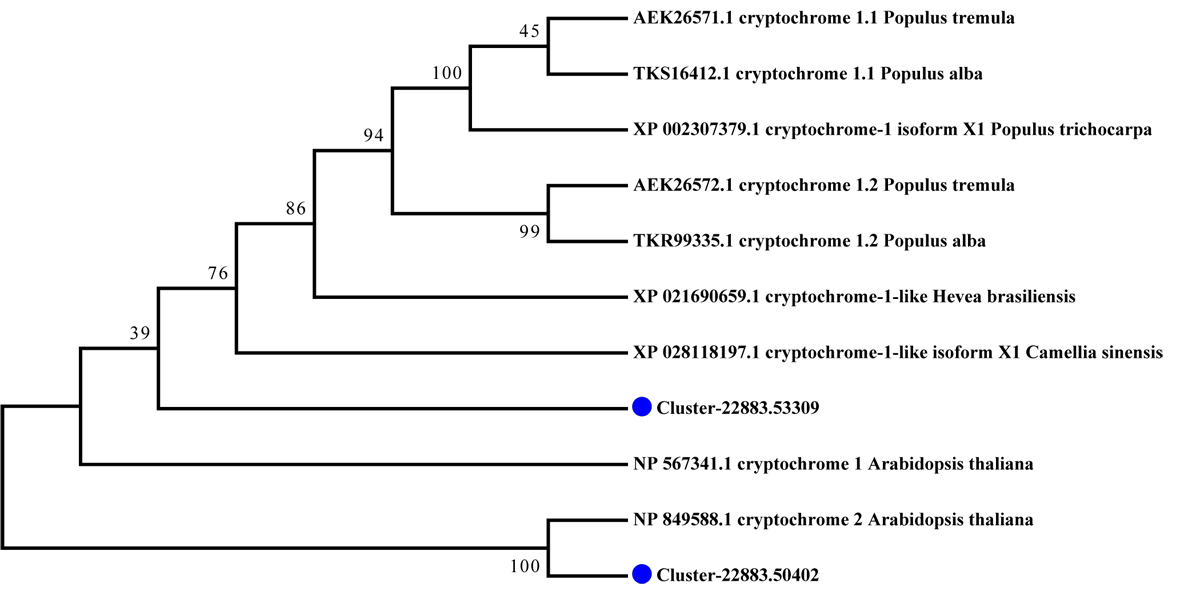


**Fig. S8a Phylogenetic tree constructed on the basis of 11 amino acid sequences belonging to Cryptochromes (Cry) family proteins.** Bootstrap values are displayed as percentages (1000 replicates) at the branches. As shown in figure, the blue circles showed the putative Cry proteins from *S. glabra*, Cluster-22883.53309 was homologous to other Cry1 proteins and then clustered into Cry1 subclass. Cluster-22883.50402 exhibited high similarity to Cry2 from Arabidapsis and classified into subclass of Cry2.


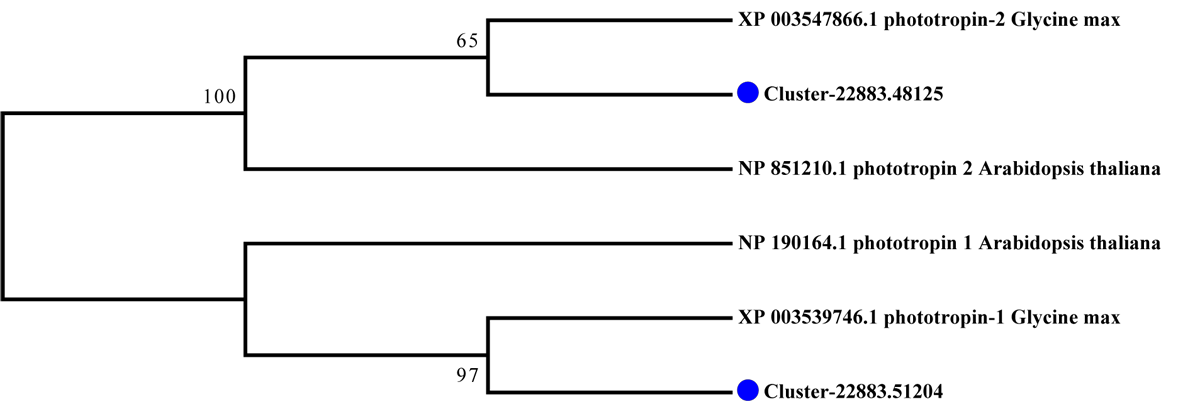


**Fig. S8b Phylogenetic tree constructed on the basis of 6 amino acid sequences belonging to Phototropin (Phot) family proteins.** Bootstrap values are displayed as percentages (1000 replicates) at the branches. As shown in figure, the blue circles showed the putative Phot proteins from *S. glabra*, Cluster-22883.48125 was homologous to other Phot2 proteins from other species. Cluster-22883.51204 exhibited high similarity to Phot1 from Arabidapsis and Glycine max and classified into subclass of Phot1.
