# Supplementary material for: Transcriptomic and metabolomic profiling reveals the effect of LED light quality on morphological traits, and phenylpropanoid-derived compounds accumulation in Sarcandra glabra seedlings
Source: BMC Plant Biol. 2020 Oct 15;20:476. doi: 10.1186/s12870-020-02685-w (PMC7574309; doi:10.1186/s12870-020-02685-w)
Supplement: Supplementary file 16 — Additional file 16: Figure S9. Phylogenetic tree constructed on the basis of 8 amino acid sequences belonging to Phenylalanine ammonia lyase (PAL) family proteins (Fig. S9a); Phylogenetic tree constructed on the basis of 8 amino acid sequences belonging to p-coumaroyl coenzyme A ligase (4-coumaroyl CoA ligase, 4CL) family proteins (Fig. S9b). [file 12870_2020_2685_MOESM16_ESM.doc]

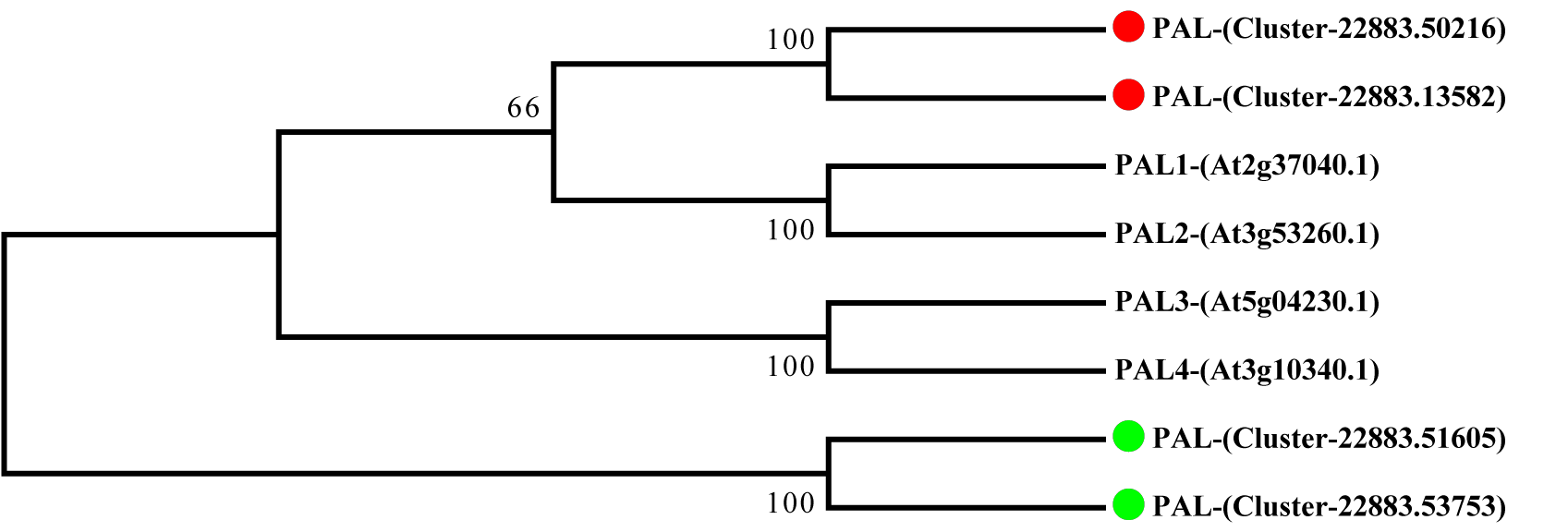


**Fig. S9a Phylogenetic tree constructed on the basis of 8 amino acid sequences belonging to phenylalanine ammonia-lyase (PAL) family proteins.** Bootstrap values are displayed as percentages (1000 replicates) at the branches. As shown in figure, the red and green circles showed the putative PAL proteins from *S. glabra*, Cluster-22883.50126 and Cluster-22883.13528 were homologous to PAL1 and PAL2 proteins from *A. thaliana****,*** while Cluster-22883.51605 and Cluster-22883.53753 were not predicted specific function.


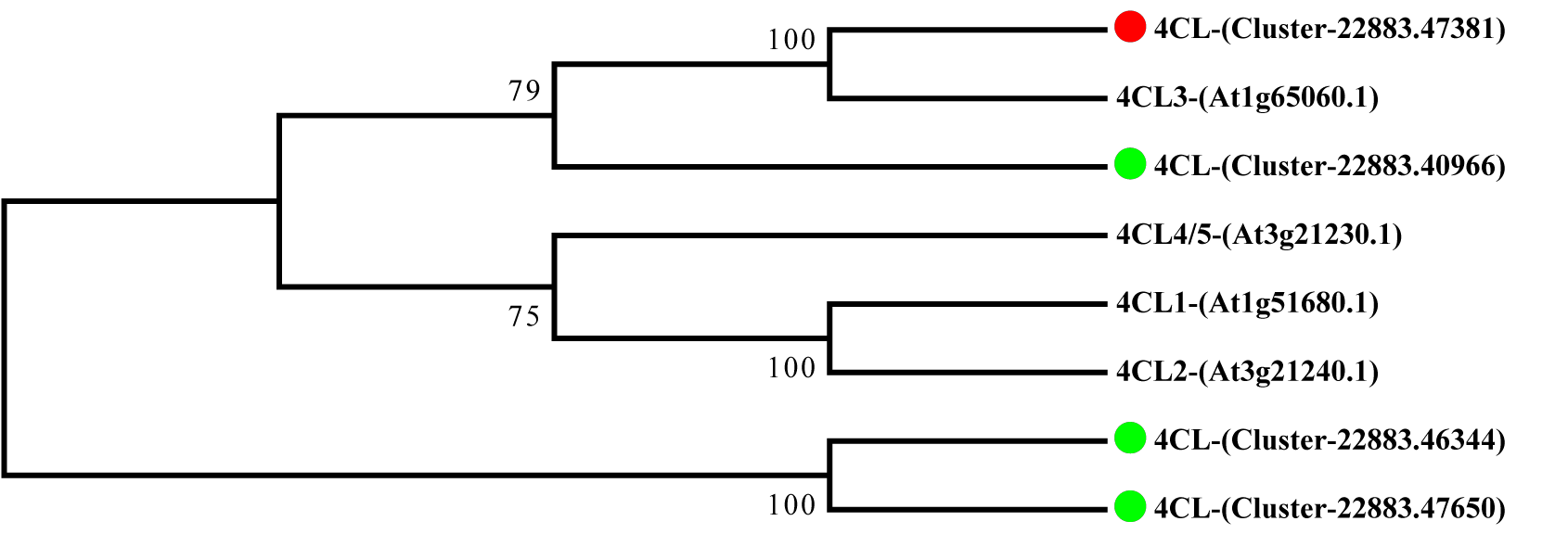


**Fig. S9b Phylogenetic tree constructed on the basis of 8 amino acid sequences belonging to p-coumaroyl coenzyme A ligase (4-coumaroyl CoA ligase, 4CL).** Bootstrap values are displayed as percentages (1000 replicates) at the branches. As shown in figure, the red and green circles showed the putative 4CL proteins from *S. glabra*, Cluster-22883.47381 and Cluster-22883.40966 were homologous to 4CL3 proteins from *A. thaliana,* while Cluster-22883.46344 and Cluster-22883.47650 were not predicted specific function.
