# Supplementary material for: Transcriptomic and metabolomic profiling reveals the effect of LED light quality on morphological traits, and phenylpropanoid-derived compounds accumulation in Sarcandra glabra seedlings
Source: BMC Plant Biol. 2020 Oct 15;20:476. doi: 10.1186/s12870-020-02685-w (PMC7574309; doi:10.1186/s12870-020-02685-w)
Supplement: Supplementary file 17 — Additional file 17: Table S8. Gene-specific primers for qRT-PCR. [file 12870_2020_2685_MOESM17_ESM.doc]

| Name of gene | Foward /Reverse Primer (5'-3') | product size (bp) |
| --- | --- | --- |
| Sg(bHLH)45 | F:TGCGACAAAAGGAACCATCC; R:ACCGAAACGCTTGCGTAATG | 86 |
| Sg(bHLH)43 | F:ATCGCCATGAAACCCAACTG; R:TCGCGTCTTTAGAGGTTTGC | 108 |
| Sg(bHLH)12 | F:AGCGCAAACCATGTTCTAGC; R:TCACAAATGGGACCAACGAC | 88 |
| Sg(bHLH)13 | F:AGGCTTTCTGCTGCATTGTC; R:GCCAGATGTGGTGACCATTTTC | 133 |
| Sg(bHLH)37 | F:TTCGACTGCCATAGTGAGGATG; R:ACGTGCTCTTCTTGCTGATG | 87 |
| SgMYB27 | F:TGACGCTGACATGTCAAAGC; R:ACTGCGAAGTTGACAAAGCC | 78 |
| SgMYB6 | F:TAGCTTGGCAGGTCTCTTCATG; R:TTGTGAAGGCCCATTTTCCC | 132 |
| SgMYB32 | F:TACATGGCTTGTTGGGCAAC; R:TGGTCGTCGTCGTGATTTTC | 120 |
| SgMYB38 | F:TCAAGAGGGTCAACAGTGATGG; R:TTTCATGGCCAACCTGCTTG | 110 |
| SgMYB53 | F:AAGCAGCAAGAGAACAAGGG; R:AAAGACTCCAGAGGTCAACGTC | 84 |
| FLS | F:ATGGCCTGTATTCTGTTCGC; R:TCGCCGGATTTTCATTGTCG | 80 |
| 4CL | F:AAGTTCCGGTCGCATTTGTG; R:TGCAGGGGATTTTGGAATGG | 143 |
| PAL | F:ACTTACTCAAGGTCGTCGATCG; R:TCGCCATTGACCAAAGCATG | 124 |
| C4H | F:TGCTTGCCAACAATCCGAAC; R:TTCGCCTCAACCTTTGCTTC | 88 |
| HCT | F:TGTTGCCGTTGCTGACAAAC; R:TCCTCGTGCTTGAAAGTGAC | 84 |
| RT | F:GACACTCCGACAGCACAAGA; R:TCATGGAGGCGAAAGGCAAT | 83 |
| CHS | F:TACAGGTGAAGGGTTGGATTGG; R:TGCAGGACAACGGTTTCAAC | 75 |
| C3H | F:ATCAATGTGTGGGCCATTGC; R:ATGGCAAAAGGCGGAAATCG | 121 |
| CHI | F:ATCGCGACCATCTCCAAAAC; R:AAACACCAAACGCGTACACG | 133 |
| F3H | F:GCCGAAAAATTGTGGAAGCG; R:GCAGGTAACGCGAAAAACTC | 121 |
| F3'H | F:TGCATGCCAGGTTTGATAGC; R:AACTTGCCACCATTGCCATC | 139 |
| DFR | F:GAGAGCTTTTGGACGGATGTTG; R:TCGGCCAATGTTTTCGAGAC | 83 |
| ANS | F:AAGATAGCGTCGTCCTCAAGC; R:AATGTGCTGCGAGAATGTGC | 89 |
| UFGT | F:TTTGAGTAAGGCGGGTTTGC; R:TGTGGCATGCTCTTTGAGTG | 103 |
| CAC | F:TCCGACAAATTGGAGGTTGC; R:TGCTGCTGACAACAATCACG | 75 |

**Table S8 Gene-specific primers for qRT-PCR**
